# Transcription of putative tonoplast transporters in response to glyphosate and paraquat stress in *Conyza bonariensis* and *Conyza canadensis* and selection of reference genes for qRT-PCR

**DOI:** 10.1371/journal.pone.0180794

**Published:** 2017-07-10

**Authors:** Marcelo L. Moretti, Rocio Alárcon-Reverte, Stephen Pearce, Sarah Morran, Bradley D. Hanson

**Affiliations:** 1 Department of Plant Sciences, University of California, Davis, California, United States of America; 2 Department of Horticulture, Oregon State University, Corvallis, Oregon, United States of America; 3 Department of Soil and Crop Sciences, Colorado State University, Fort Collins, Colorado, United States of America; University of Illinois at Urbana-Champaign, UNITED STATES

## Abstract

Herbicide resistance is a challenge for modern agriculture further complicated by cases of resistance to multiple herbicides. *Conyza bonariensis* and *Conyza canadensis* are invasive weeds of field crops, orchards, and non-cropped areas in many parts of the world. In California, USA, *Conyza* populations resistant to the herbicides glyphosate and paraquat have recently been described. Although the mechanism conferring resistance to glyphosate and paraquat in these species was not elucidated, reduced translocation of these herbicides was observed under experimental conditions in both species. Glyphosate and paraquat resistance associated with reduced translocation are hypothesized to be a result of sequestration of herbicides into the vacuole, with the possible involvement of over-expression of genes encoding tonoplast transporters of ABC-transporter families in cases of glyphosate resistance or cationic amino acid transporters (CAT) in cases of paraquat resistance. However, gene expression in response to herbicide treatment has not been studied in glyphosate and paraquat resistant populations. In the current study, we evaluated the transcript levels of genes possibly involved in resistance using real-time PCR. First, we evaluated eight candidate reference genes following herbicide treatment and selected three genes that exhibited stable expression profiles; *ACTIN*, *HEAT-SHOCK-PROTEIN-70*, and *CYCLOPHILIN*. The reference genes identified here can be used for further studies related to plant-herbicide interactions. We used these reference genes to assay the transcript levels of *EPSPS*, ABC transporters, and *CAT* in response to herbicide treatment in susceptible and resistant *Conyza* spp. lines. No transcription changes were observed in *EPSPS* or *CAT* genes after glyphosate or paraquat treatment, suggesting that these genes are not involved in the resistance mechanism. Transcription of the two ABC transporter genes increased following glyphosate treatment in all *Conyza* spp. lines. Transcription of ABC transporters also increased after paraquat treatment in all three lines of *C*. *bonariensis*. However, in *C*. *canadensis*, paraquat treatment increased transcription of only one ABC transporter gene in the susceptible line. The increase in transcription of ABC transporters after herbicide treatment is likely a stress response based on similar response observed across all *Conyza* lines regardless of resistance or sensitivity to glyphosate or paraquat, thus these genes do not appear to be directly involved in the mechanism of resistance in *Conyza* spp.

## Introduction

Herbicide resistance is widespread in modern agriculture and challenges the long-term sustainability of herbicide use [1]. Although many herbicides are commercially available, the market is dominated by a small number of widely used chemicals [2, 3]. Two of the most extensively used herbicides are glyphosate [4] and paraquat [5]. The repeated use of these herbicides has selected for the evolution in many weed species of resistance to individual herbicide modes of action and, increasingly, to multiple herbicides. Reports of dual resistance to both glyphosate and paraquat in weedy species are limited, but cases in *Conyza bonariensis* and *Conyza canadensis* were recently reported in several locations in California, USA [6, 7]. Members of the *Conyza* genus are listed among the most problematic herbicide-resistant weeds globally based on number of countries where they are present, number of herbicide modes of action with documented resistance, and estimated infested sites and cropping systems [8, 9]; these weeds affect corn, soybean, vineyards, orchards, and non-cropped areas across five continents [10]. Due to the agronomic and economic importance of *C*. *bonariensis* and *C*. *canadensis* in the U.S. and other parts of world, there is an urgent need to understand the mechanisms of glyphosate and paraquat resistance to help combat this threat.

Glyphosate is a mobile, foliar-applied herbicide which inhibits the enzyme 5-enolpyruvylshikimate-3-phosphate synthase (EPSPS) [11]. Glyphosate resistance has been reported in several weed species with various mechanisms documented to date [12]. In *Conyza* spp., reduced translocation is the predominant resistance mechanism [12], which is associated with sequestration of the herbicide into the vacuole, thus neutralizing its activity [13]. Vacuole sequestration of glyphosate may be dependent on the activity of tonoplast-localized transporters [12, 13]. Although the specific transporters involved in glyphosate resistance in *Conyza* spp. have not been identified, two members of the ABC transporter super-family—*M10* and *M11*—have been identified as candidate genes [14, 15]. These putative transporters are homologous to the *Arabidopsis* genes *ABCC10* and *ABCC8*, respectively, which encode members of the ABCC sub-group of tonoplast-localized ABC transporters.[16]. In *C*. *canadensis*, *M10* and *M11* transcript levels exhibit a 45- to 300-fold increase in response to glyphosate treatment, with the response being greatest in glyphosate-resistant plants [14]. The increase in transcription of *M10* and *M11* in this species was reported to be synchronized with an increase in *EPSPS* transcription and affected by environmental conditions [15, 17]

Paraquat is a foliar-applied herbicide with limited systemic mobility in the plant, which targets the chloroplasts, where it acts as a photosystem I electron acceptor generating reactive oxygen species [18]. Paraquat resistance in *Conyza* spp. and other weed species is also associated with reduced translocation of the herbicide and related to vacuolar sequestration [18–21]. Although no specific transporter involved in paraquat sequestration into the vacuole has been identified, a study with *C*. *canadensis* suggested the involvement of a cationic amino acid transporter (CAT), [22]. In *Arabidopsis*, both *CAT2* (AT1G58030) and *CAT4* (AT3G03720) encode CATs which are primarily localized to the tonoplast [23], making them appropriate candidates for the vacuolar sequestration of paraquat.

In glyphosate-paraquat-resistant (GPR) *Conyza spp*., reduced translocation of glyphosate and paraquat was observed in both *C*. *bonariensis* and *C*. *canadensis* and paraquat resistance was always associated with glyphosate resistance [24]. However, this previous work did not investigate the molecular aspects of resistance. Characterizing the transcriptional responses of candidate genes in response to glyphosate and paraquat treatment is one approach that could shed light on the mechanism(s) of resistance to these herbicides in *Conyza* spp. A pre-requisite for the accurate determination of target gene transcript levels in qRT-PCR studies is the selection of stably-expressed reference genes [25, 26]. In model plant species, large, publicly-available databases describing temporal and spatial gene expression profiles facilitate the selection of reference genes [27]. However, non-model species such as *Conyza* lack these resources, meaning the selection and validation of stable reference genes must be performed empirically.

In the current study, we report the selection and validation of stably-expressed reference qRT-PCR assays for expression studies in *Conyza* spp. under glyphosate or paraquat stress. Using these reference genes, we evaluated the transcriptional profiles of candidate transporter genes in response to herbicide treatment to determine their involvement in the herbicide resistance mechanism.

## Materials and methods

### Plant material and growth conditions

Single seed descendants of previously characterized biotypes of three *C*. *bonariensis* and three *C*. *canadensis* were selected for these studies, hereafter referred to as lines. The phenotypes of these lines were: glyphosate and paraquat susceptible (GPS), glyphosate-resistant (GR), or glyphosate- and paraquat-resistant (GPR) [24]. The resistant lines of both species demonstrated similar reductions in the translocation of glyphosate (GR) or glyphosate and paraquat (GPR) from the treated leaf. All lines were self-pollinated for at least three generations and recurrently selected for the phenotype (GR, GPR, or GPS) to ensure homogeneous response. Seeds were placed in flats filled with commercial potting mix (Sun Gro Horticulture, Vancouver, BC, CAN) and kept in growth chambers (PGR15 Conviron, Manitoba, Canada) at 28°C/22°C day/night temperatures with 16 hours of light and 50% relative humidity. These conditions were maintained throughout the experiments. After germination, individual seedlings were transplanted into 0.5 L-pots and kept in the growth chamber. Plants were grown for 28 days before herbicide treatments were imposed on 5- to 7-leaf stage *C*. *bonariensis* or 8- to 10-leaf stage *C*. *canadensis* plants.

### Herbicide treatment and sample collection

Response to glyphosate and paraquat was evaluated in independent experiments. In each experiment, three lines (GPS, GR, and GPR) of each species were either treated with the herbicide or left untreated. Six biological replicates were used for each treatment and line combination resulting in 72 samples per experiment. To evaluate the response to glyphosate, plants were treated with 29.56 mM of glyphosate acid, equivalent to a field rate of 1 kg of glyphosate acid ha^-1^ (glyphosate, Roundup PowerMax, 540 g acid equivalent L^-1^, Monsanto Co, St Louis, MO, USA). To evaluate the response to paraquat, plants were treated with 9.7 mM of the paraquat cation, equivalent to a field rate of 0.5 kg of paraquat ha^-1^ (Gramoxone Inteon^®^, 240 g ai L^-1^, Syngenta Crop Protection Inc., Greensboro, NC, USA). Herbicide solutions also included non-ionic surfactant (R-11^®^, 90% v/v, Wilbur-Ellis, Aurora, CO) at 0.25% v/v and ammonium sulfate at 1% w/v following label recommendations. Herbicide treatments were applied to foliage using a single-nozzle spray-chamber calibrated to deliver the equivalent of 200 L ha^-1^. Application occurred in the mornings between 8 and 10 am.

Leaf tissues were collected 24 hours after glyphosate application and 6 hours after paraquat application [14]. Although paraquat causes rapid desiccation of leaves in susceptible plants, functional activity of leaves was confirmed using maximum quantum of photosystem II within 6 hours after treatment in a preliminary study (S1 Fig), and by other researchers [22]. The sample collections consisted of excising the youngest, fully-expanded leaf at the base of the rosette for each biological replicate, and immediately placing it in liquid nitrogen. Samples were stored at -80°C until further analysis.

### RNA extraction and cDNA synthesis

Total RNA was extracted using RNeasy Plant mini kit (Qiagen, Valencia, CA, USA) following the manufacturer’s protocol. RNA concentration and quality was assessed by measuring absorbance at 230, 260, and 280 nm using a ND-1000 spectrophotometer (NanoDrop Technologies, Wilmington, DE, USA). Sample quality was considered adequate when the absorbance ratio of A260/A280 was between 1.8 to 2 and the ratio of A260/A230 was greater than 2 [28]. RNA integrity was assessed by electrophoresis using a 2% agarose gel. cDNA synthesis was performed using 1 μg of template RNA using QuantiTect Reverse Transcription Kit (Qiagen, Valencia, CA, USA), including treatment with gDNA wipe-out buffer to mitigate DNA contamination, as recommended by the manufacturer.

### Quantitative reverse-transcription polymerase chain reaction (qRT-PCR) analysis

Real-time PCR was conducted using the 7500 Fast Real-Time PCR system (Applied Biosystems, Foster City, CA, USA). Reactions were performed in 20 μL volumes comprised of 10 μL of Fast SYBR Green master mix (2X) (Applied Biosystems, Foster City, CA, USA), 200 nM of each primer and 20 ng of cDNA template. The reaction conditions consisted of single cycle of 20 s at 95°C followed by 40 cycles of two steps: first 3 s at 95°C followed by 30 s at 60°C.

### Reference genes and target genes selection and validation for qRT-PCR

Eight genes commonly used as qRT-PCR reference genes in *Arabidopsis thaliana* were evaluated as candidate reference genes in *C*. *bonariensis* and *C*. *canadensis* [29]. The *Arabidopsis thaliana* nucleotide coding sequences of candidate reference genes were used as queries in a BLASTN search optimized for discontinuous megablast against the *Conyza canadensis* genome [30] from the NCBI assembly database [31]. The gene names, description, and details of the BLAST search are presented in Table 1.

**Table 1:** Candidate qRT-PCR reference and target genes used in this study. *Arabidopsis thaliana* coding sequences were used to query *C*. *canadensis* genome assembly. The identity match (%) and e-value of the selected BLAST hit are listed.

Gene description ^a^	*Arabidopsis* reference ^b^	Homologous locus	*C*. *canadensis* identifier ^c^	ID (%)^d^	e-value ^e^
**Reference genes**					
*ACTIN 7 (ACT7)*	NM_121018.3	AT5G09810	JSWR01015158.1	85	9e^-178^
*TUBULIN ALPHA-6 (TUA6)*	NM_117582.3	AT4G14960	JSWR01005675.1	83	0.0
*EUKARYOTIC ELONGATION FACTOR 1- α (eEF-1α)*	NM_125432.3	AT5G60390	JSWR01002849.1	86	0.0
*EUKARYOTIC INITIATION FACTOR 4α (eIF-4α)*	NM_101261.2	AT1G13950	JSWR01008862.1	83	6e^-30^
*GLYCERALDEHYDE-3-PHOSPHATE DEHYDROGENASE (GAPDH)*	NM_113576.3	AT3G26650	JSWR01003254.1	78	1e^-112^
*HEAT SHOCK PROTEIN 70–4 (HSP70)*	NM_112093.2	AT3G12580	JSWR01000228.1	80	3e^-39^
*UBIQUITIN 3 (UBQ3)*	NM_115119.3	AT3G52590	JSWR01017475.1	84	1e^-26^
*CYCLOPHILIN 5 (CYP5)*	NM_119653.3	AT4G34870	JSWR01003450.1	76	4e^-104^
**Target genes**					
*ABC-C FAMILY MRP10 (M10)*	NM_116135.2	AT3G62700	JSWR01002397.1	76	3e^-57^
	NM_130347.4	AT2G47800		77	
*ABC-C FAMILY MRP8 (M11)*	NM_112148.3	AT3G13090	JSWR01014711.1	67	4e^-105^
*CATIONIC AMINO ACID TRANSPORTER 4 (CAT4)*	NM_111243.7	AT3G03720	JSWR01002601.1	77	2e^-37^
	NM_179491.2	AT1G58030		83	
*5-ENOL-PYRUVYLSHIKIMATE-3-PHOSPHATE SYNTHASE (EPSPS)*	NM_130093.2	AT2G45300	AY545666; JSWR01004464.1	78	7e^-41^
			AY545667; JSWR01002887.1	77	3e^-39^
			AY545668; JSWR01015937.1	71	2e^-23^

^a^ Gene nomenclature followed *Arabidopsis thaliana* genes.

^b^ *Arabidopsis thaliana* genome sequence identifier of reference coding sequence used in BLASTN.

^c^ Accession number of sequence from *C*. *canadensis* assembly most similar to *Arabidopsis* reference

^d^ Sequence identity similarity as percent of sequence similarity between query and reference sequences.

^e^ Number of hits with similar sequence present in the database by chance.

Homolog detection was performed using a reciprocal BLAST search as described previously [32]. The *C*. *canadensis* genomic sequences most similar to the *A*. *thaliana* reference coding sequences were used to design qRT-PCR primers. Primer design was performed using the Primer-BLAST tool based on Primer 3 [33, 34]. The primers used for the *ACTIN* genes were previously published by Peng et al. [14]. The primers for *EPSPS* were designed to amplify conserved regions of all three known copies of the *EPSPS* gene from *C*. *canadensis* [35]. New primers were designed for *M10* and *M11* using as templates the direct sequences of the cDNA regions amplified by the primers originally developed by Peng et al. [14] (S1 Appendix). The primers designed used to amplify cDNA fragment were: *M10* Forward (5’-TTGGCTCAACTTCGTGGTATCGGG-3′) and reverse (5′-CCAAGAAATTCCAAGCGGAACCCT-3′) with an amplified fragment of 253 bp; *M11* Forward (5′-ATGCTGTCTTCTTTTACCTTTGC-3′) and reverse (5′-CGACTTCCCACTACCAGTTCTTC-3′) with an amplified fragment of 393 bp [14].

The thermocycle conditions were as follows: one initial cycle at 95°C for 2 min, followed by 35 cycles of 95°C for 30 seconds, 60°C for 30 seconds, 72°C for 2 minutes. The reaction was terminated with an extension cycle at 72°C for 7 minutes.

Primer pairs were selected based on low predicted likelihood of forming secondary structures (including self-dimerization, hairpin, and self-annealing), a prediction made by the OligoCalc online tool [36]. Primer specificities were tested by performing PCR using cDNA of both *Conyza* spp. as templates and running the amplified product on a 2% agarose gel. We also analyzed the shape of the dissociation curve to confirm each product had a single peak melting curve (S2 Fig). Amplification efficiencies were calculated using the slope of Ct values derived from a four-fold cDNA dilution series. Efficiencies were calculated using the formula: efficiency (%) = (10^(-1/slope)^-1)*100 and ranged from 84 to 99% (Table 2).

**Table 2:** Primer sequence and amplicon size of candidate reference genes and target genes evaluated. Primer amplification efficiencies were calculated for each of the *Conyza* spp.

Gene ^a^	Sequences		Amplicon size (bp)	Primer efficiency (%)	
	Forward (5’ to 3’)	Reverse (5’ to 3’)		*C*. *bonariensis*	*C*. *canadensis*
*ACT7* ^b^	GTGGTTCAACTATGTTCCCTG	CTTAGAAGCATTTCCTGTGG	228	95.7	89.8
*TUA6*	TGGCTCCACAACAGAGGTAG	AACTGGTTCCGGTCTTGGAT	121	93.5	95.7
*eEF-1α*	AACCACCATACCAGGCTTGA	AGCCCAAGAGACCATCAGAC	125	94.7	99.8
*eIF-5a*	TTTCTACCTCCAAGACCGGC	CAATTGTGGGATGAGGGCAC	109	94.9	90.9
*GAPDH*	GCCAAATCAACCACCCTCTG	AGTGATGTCTCGTCAACCGT	119	97.9	93.8
*HSP70*	CTTGCAAAGCTCCTTTCCGT	AAGTGTATGGAGCCCGTTGA	150	99.5	84.9
*UBQ3*	TATGCTCGTCTCCACCCAAG	GCCACACTTCTTCTTCCTGC	51	95.6	95.6
*CYP5*	ACAGGCTTAGAGGTGGTTCC	TGGGAAGCATGTGGTGTTTG	99	95.4	94.5
*CAT4*	AAGAAAAGGCCAGAGCAGGA	TTCTTGTGGGTACGGTTGCT	54	91.20	91.94
*M10*	GGGGCTATTACCTTGCAACA	GGTCATAACCCCTGAGATGC	104	82.05	85.55
*M11*	TGGACTTTAACCAACCTTGAAAA	CCTGTGACGGCCACTGAT	127	95.84	97.79
*EPSPS* ^b^	TTACTTCTTAGCTGGTGCTG	GGCATTTTGTTCATGTTCACATC	220	95.18	94.73

^a^ Gene nomenclature followed *Arabidopsis thaliana* genes.

^b^ Primer sequences previously published [14].

### Reference gene stability

The cDNA samples from three biological replicates of each line and treatment combination were selected to analyze the stability of the candidate genes’ transcript levels across species, genotypes and herbicide treatments. Selection of candidate genes was performed independently for each herbicide and species. Evaluation of candidate genes was based on the number of cycles required for the fluorescence signal to surpass the threshold level, or cycle threshold (Ct). Ct values were assigned by a built-in algorithm in the qRT-PCR system using default settings. Ct values were assessed visually and no manual adjustments were necessary. Presence of outliers was tested using the Grubb’s test (p<0.05), and outliers were removed prior to further analysis [37]. Ct means and standard deviations for evaluated reference genes are available in S1 Dataset.

Many algorithms are available to evaluate gene stability, and the final outcome of gene stability is comparable among many of these algorithms [38]. Candidate gene stability was evaluated using the NormFinder algorithm because this algorithm can evaluate different sample groups and separate intra- versus inter-group variation [39]. The variation is summarized in a single stability parameter with smaller values indicating greater transcription stability. NormFinder stability parameters were calculated for each treatment and species and genes were ranked based on stability. A consensus ranking was calculated using the Monte Carlo algorithm based on the Spearman’s distances across rankings performed by RankAggreg package in R [40]. The three most stable genes were selected as reference genes to assay the transcript levels of target genes.

### Expression of ABC-transporters, *CAT*, and *EPSPS* genes

Candidate gene transcript levels were quantified in the youngest fully-expanded leaf tissue 24 hours after treatment with glyphosate or six hours after treatment with paraquat. The genes *ACT7*, *CYP5*, and *HPS70* were used as reference genes and the geometric means of all three genes was used for normalization. For each line and herbicide, target gene transcript levels were compared between treated and untreated plants using six biological replicates. Ct means and standard deviations for evaluated target genes are available on S1 Dataset. Transcription of the four target genes was compared using the 2^-ΔΔCT^ method for each gene and line [41]. A t-test (*p* <0.05) was used to compare the effect of herbicide treatment on transcription of target genes within line. To compare the basal expression level of target genes among lines before herbicide treatment, we used the 2^-ΔCT^ method [42]. These expression values are presented relative to the geometric mean of the three reference genes *ACT7*, *CYP5*, and *HPS70* for each line. A t-test (*p* <0.05) was used to make pair-wise comparison of lines for each species and target gene combination.

## Results

### Validation of qRT-PCR assays for reference and target genes in *Conyza* spp.

To develop stable qRT-PCR reference gene assays for herbicide treatment studies in *Conyza* spp. we selected eight candidate genes previously used in qRT-PCR studies in *Arabidopsis* [29]. The coding sequences for each gene was queried against the *Conyza canadensis* genome assembly and three pairs of qRT-PCR primers were designed. Based on several quality parameters (S2 Fig) and affinity for both species, the best primer pair was selected (Table 2).

We evaluated the expression stability of each candidate reference gene using cDNA samples from the leaves of three lines (GPR, GR, and GPS) of both species from untreated plants and plants treated with either glyphosate or paraquat. In response to glyphosate treatment, the three most stable genes were *ACT7*, *HPS70*, and *CYP5* in *C*. *bonariensis*, and *HPS70*, *CYP5*, and *ACT7* in *C*. *canadensis* (Table 3). In response to paraquat treatment, the three most stable genes were *CYP5*, *ACT7*, and *HPS70* in *C*. *bonariensis*, and *CYP5*, *ACT7*, and *HPS70* in *C*. *canadensis* (Table 3).

**Table 3:** Transcription stability values and stability ranking of candidate reference genes calculated using the NormFinder algorithm. Transcription was evaluated on young leaves of *Conyza bonariensis* and *Conyza canadensis* lines after glyphosate or paraquat treatments.

Gene name	Glyphosate				Paraquat			
	*C*. *bonariensis*		*C*. *canadensis*		*C*. *bonariensis*		*C*. *canadensis*	
	Stability	Rank	Stability	Rank	Stability	Rank	Stability	Rank
*ACT7*	0.017	1	0.017	3	0.011	2	0.02	2
*TUA6*	0.037	6	0.020	5	0.035	8	0.030	6
*CYP5*	0.022	3	0.015	2	0.008	1	0.01	1
*eEF- 1α*	0.030	4	0.022	6	0.012	5	0.020	4
*eIF-5a*	0.030	5	0.020	4	0.013	6	0.030	5
*GAPDH*	0.039	7	0.041	8	0.016	7	0.050	8
*HPS70*	0.018	2	0.010	1	0.011	3	0.020	3
*UBQ6*	0.042	8	0.031	7	0.011	4	0.040	7

**Fig 1:**
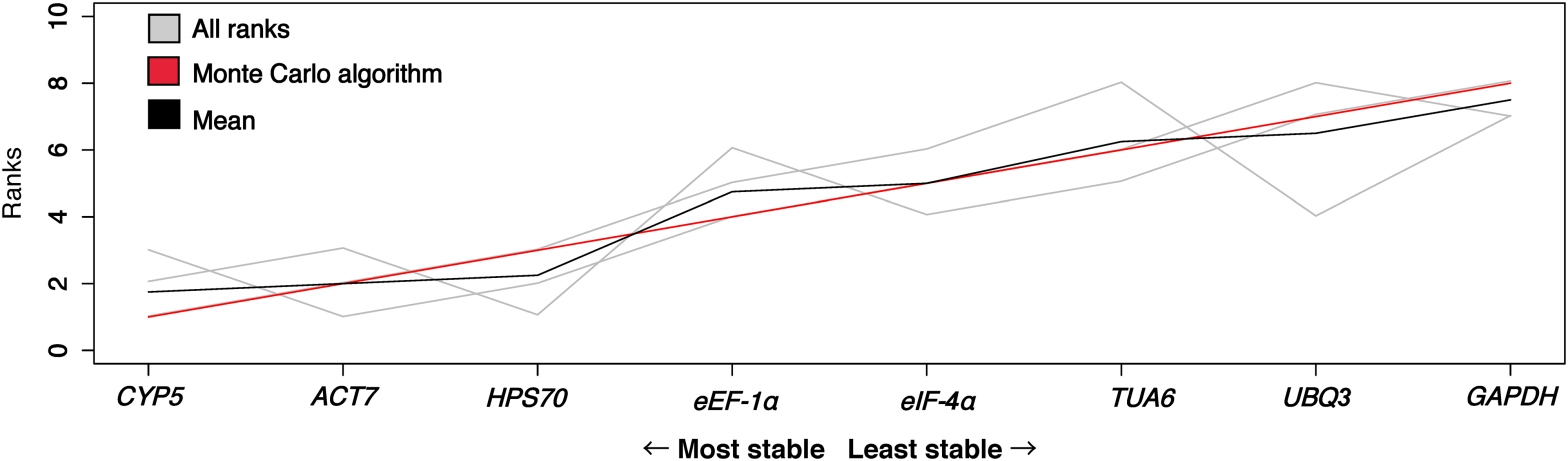
Consensus ranking of reference gene stability in *Conyza* spp. using Monte Carlo algorithm based on Spearman distances. Genes are ordered by decreasing stability values based on their rankings (grey lines) calculated by Normfinder in *C*. *bonariensis* and *C*. *canadensis* treated with glyphosate or paraquat. The mean ranking is indicated by the black line, and the consensus ranking computed by Cross-Entropy (CE) Monte Carlo algorithm is indicated by the red line. The genes cyclophilin 5 (*CYP5*), actin 7 (*ACT7*), and heat-shock protein 70 (*HPS70*) were the three most stable genes.

The consensus ranking of gene stability identified the genes *CYP5*, *ACT7*, and *HPS70* as the most stable across species and herbicide treatments (Fig 1), and therefore these genes were used as reference genes in the subsequent experiments.

### Expression of ABC-transporters, *CAT*, and *EPSPS* genes in response to herbicide treatment

**Fig 2:**
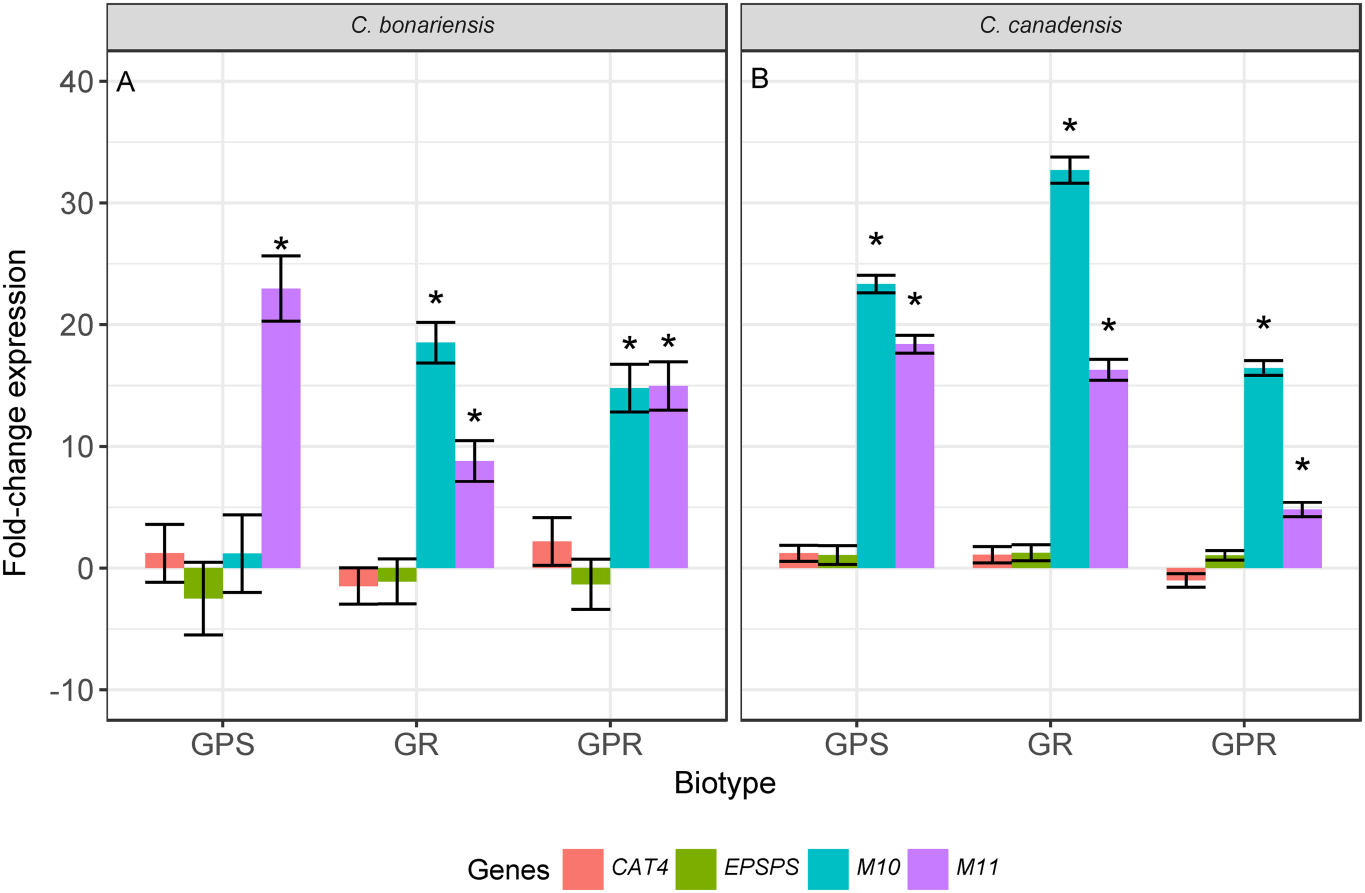
Transcripts levels of *EPSPS*, *CAT4*, and two ABC transporters in glyphosate treated plants relative to untreated plants in lines of *Conyza bonariensis* (A) and *Conyza canade*nsis (B). Lines were glyphosate-paraquat-susceptible (GPS), glyphosate-resistant (GR), and glyphosate-paraquat-resistant (GPR). Transcript levels were measured 24 h after glyphosate treatment and normalized using the geometric mean of actin (*ACT*), heat-shock-protein (*HPS70*), and cyclophilin 5 (*CYP5*) genes within each line. Significant differences (*p* <0.05) in transcript levels between treated and untreated plants indicated by *.

We next analyzed the transcript levels of four target genes in response to herbicide treatment, which were selected based on previous reports suggesting their involvement in glyphosate or paraquat resistance in *Conyza* spp. [14, 22]. Glyphosate treatment increased transcript levels of the putative ABC-transporter gene *M10* by 18-fold and 14-fold in the GR and GPR lines of *C*. *bonariensis*, respectively (*p* <0.05, Fig 2A). Transcript levels of *M11* were also significantly increased following glyphosate treatment in all *C*. *bonariensis* lines (8- to 23-fold, *p* <0.05). No significant changes (*p* >0.05) in transcript levels of *CAT4* or *EPSPS* were observed in response to glyphosate in *C*. *bonariensis* (Fig 2A). In *C*. *canadensis*, both ABC transporter genes were induced in response to glyphosate treatment. *M10* transcript levels increased in all lines by 16- to 32-fold (Fig 2B), whereas the increase in *M11* transcript levels was greater in GR and GPS lines (16-fold) compared to the GPR line (4-fold). Consistent with results in *C*. *bonariensis*, *CAT4* and *EPSPS* transcript levels were unaffected by glyphosate treatment (Fig 2B).

**Fig 3:**
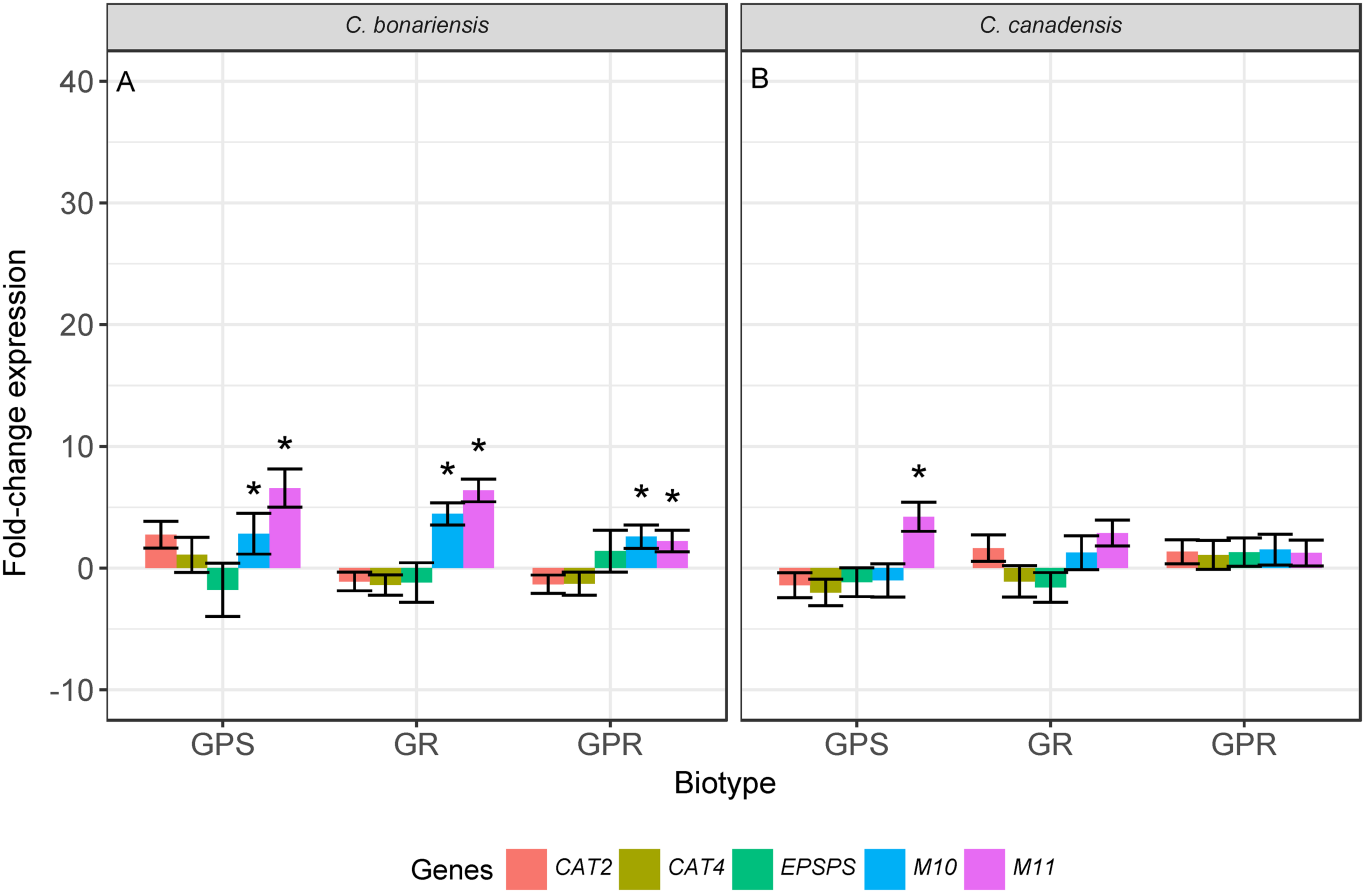
Transcript levels of *EPSPS*, *CAT4*, and two ABC transporters (*M10* and *M11*) in paraquat treated plants relative to untreated plants in lines of *Conyza bonariensis* (A) and *Conyza canade*nsis (B). Lines were glyphosate-paraquat-susceptible (GPS), glyphosate-resistant (GR), and glyphosate-paraquat-resistant (GPR). Transcript levels were measure 6 h after paraquat treatment and were normalized using the geometric mean of actin, heat-shock-protein, and cyclophilin genes within each line. Significant differences (*p* <0.05) in transcript levels between treated and untreated plants indicated by *.

An increase in the transcript levels of both ABC transporter genes was also observed in response to paraquat treatment. In *C*. *bonariensis*, transcript levels of both *M10* and *M11* were significantly increased after treatment in all lines (*p* <0.05). In *C*. *canadensis*, *M11* transcript levels in the GPS biotype were 4.2 fold higher following paraquat treatment (*p* <0.05, Fig 3B), but were not significantly different in the other biotypes. No significant differences in the transcript levels of *M10*, *EPSPS* or *CAT4* were observed in response to paraquat treatment in any of the lines of either species (Fig 3B).

**Fig 4:**
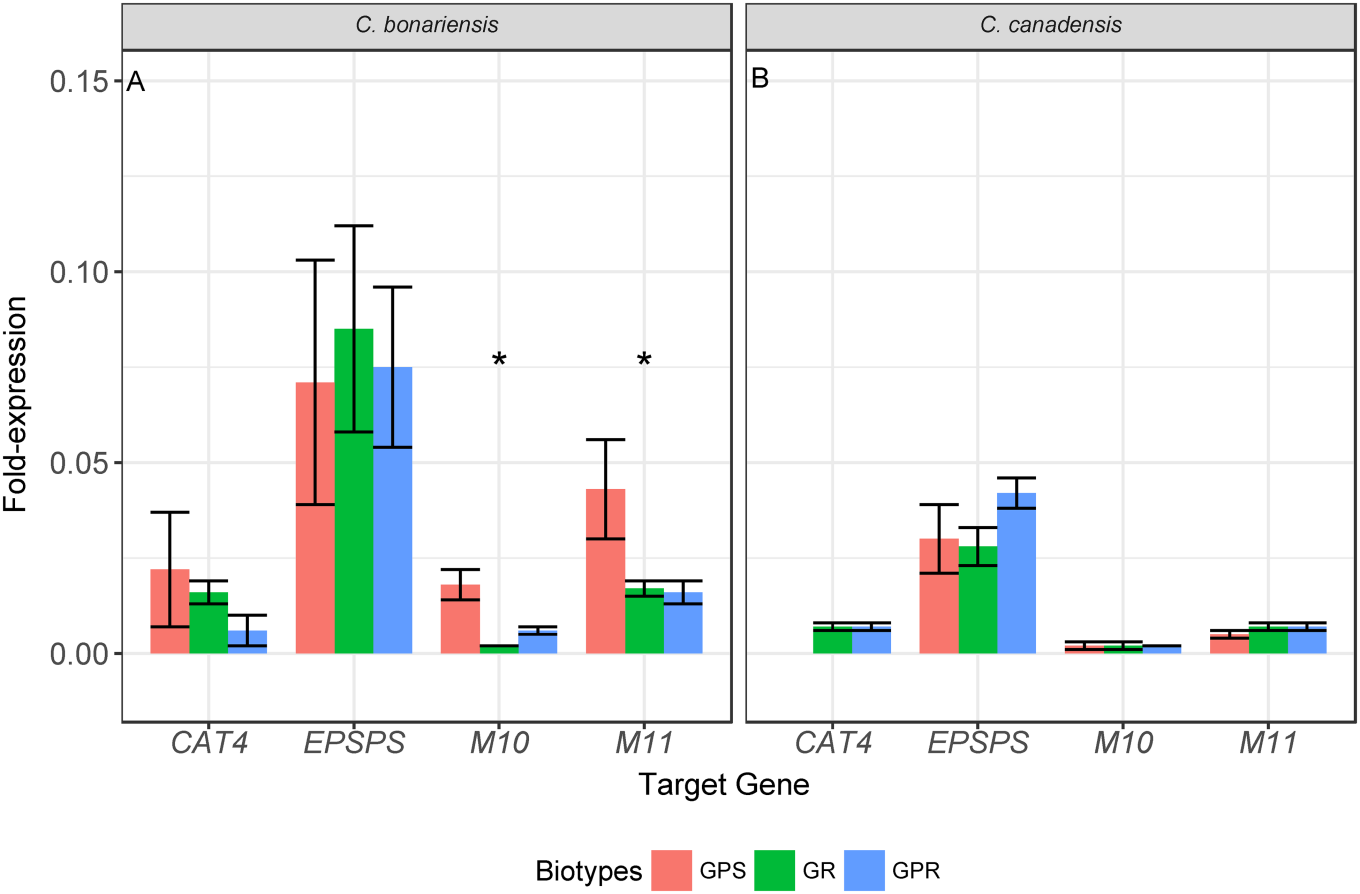
Transcript levels of *EPSPS*, *CAT4*, and two ABC transporters (*M10* and *M11*) in untreated plants of lines of *Conyza bonariensis* (A) and *Conyza canade*nsis (B). Lines were glyphosate-paraquat-susceptible (GPS), glyphosate-resistant (GR), and glyphosate-paraquat-resistant (GPR). Transcript levels were normalized using the geometric mean of actin, heat-shock-protein, and cyclophilin genes within each line. Significant differences (*p*<0.05) in transcript levels among lines are indicated by *.

To determine whether differences in resistance between lines could be attributed to differential basal transcript levels of target genes, we compared their expression in untreated plants. Among *C*. *bonariensis* lines, transcript levels of both *M10* and *M11* were significantly higher in the GPS line than in either GR or GPR lines (Fig 4A). No significant differences in *CAT4* or *EPSPS* transcript levels were found. Among *C*. *canadensis* lines, there were no significant differences in the basal transcript levels of any target gene (Fig 4B). These results suggest that differences in the basal transcript levels of the assayed target genes are unlikely to be responsible for the herbicide resistance phenotype.

## Discussion

### Validation of qRT-PCR reference genes in *Conyza* spp.

Altered transcriptional responses to treatments can provide important clues regarding the mechanism underlying biological responses, such as herbicide tolerance. Despite rapid advances in next-generation sequencing technologies, developing individual qRT-PCR assays remains the most cost effective approach to characterize changes in expression for a small number of target genes. To accurately quantify changes in transcript levels at high sensitivity using qRT-PCR, the selection of appropriate reference genes is imperative [43]. Accuracy is further improved when multiple reference genes are selected [44].

However, even commonly used reference genes can exhibit large differences in expression depending on environmental conditions, tissue sample, and organism making it important to evaluate genes prior to normalization [45]. Because target gene expression is measured relative to the expression of the reference gene, variation in the expression of the reference gene between treatments, genotypes and environments can introduce inaccuracies into expression data. Therefore, for each analysis selection of reference genes must be carefully considered based on the experimental conditions, since suitable reference genes may vary depending on a number of factors. For example, in *Alopercurus myosuroidis* Huds., a monocot weed species, *TUB*, *GADPH*, and *UBQ* were identified as the most stable reference genes after application of an acetyl-CoA carboxylase inhibitor herbicide [46]. Similarly, in *Lolium* sp., another monocot weed, *GADPH* and *UBQ* were identified as the most stable reference genes following treatment with herbicides that inhibit both acetolactate synthase or acetyl-CoA carboxylase [47]. Therefore, *TUB* was identified as a stable reference gene in *A*. *myosuroides*, but not in *Lolium* sp. [47]. In *Conyza* spp., transcriptional responses have only been evaluated in *C*. *canadensis* after glyphosate treatment. The gene *ACT* was used as reference gene, but no indication of gene stability was provided [14]. In the current study, *ACT* was identified as stable after glyphosate treatment in both *C*. *canadensis* and *C*. *bonariensis*. However, *GADPH*, *TUB*, and *UBQ* were not among the most stable genes in *Conyza* spp. (Fig 1). Likewise, *GAPDH* has been ranked unsuitable as a reference gene in bamboo [48], brachypodium [49] and pearl millet [50], while in rice [51], and soybean [52], none of these three genes were suitable as references [51, 52].

Here, we analyzed the transcript levels of eight candidate genes in *Conyza* spp. under different herbicide stress conditions, and identified three genes which showed the greatest stability across all tested conditions (*ACT7*, *HPS70*, and *CYP5*). The selected genes can be applied for further studies to investigate the mechanism of herbicide resistance in *Conyza* spp.

### Expression of *EPSPS*, *CAT* and ABC-transporter genes in response to herbicide treatment

In a previous study, we identified glyphosate and paraquat resistant lines of *C*. *bonariensis* and *C*. *canadensis* which was associated with reduced translocation of these herbicides, possibly as a result of herbicide sequestration into the vacuoles [24]. To test the hypothesis that increased expression of putative transporter genes were associated with this phenotype, we assayed the transcript levels of four target genes in response to herbicide treatment in susceptible and resistant lines.

We found that *EPSPS* transcript levels were unaffected by glyphosate or paraquat treatment in any line (Figs 2 and 3). These findings are in agreement with a previous study in glyphosate-resistant *C*. *canadensis*, which similarly showed no changes in *EPSPS* expression following herbicide application [35]. However, another study reported increased transcription of *EPSPS* after glyphosate treatment in GR *C*. *canadensis* [15]. In general, over-expression of genes encoding target enzymes is a rare mechanism of herbicide resistance in plants [1]. Increased *EPSPS* expression has been reported in other GR weed species, such as *Amaranthus palmeri* S. Watson, generally as a result of increased *EPSPS* gene copy number [12].

Many classes of transporters are involved in paraquat movement across cell membranes, including members of the Cationic Amino acid Transporter (CAT) family which are more commonly known for their role in polyamine transport [53]. Their dual role in transporting paraquat and polyamines may be a result of the similarities between these molecules [53, 54]. *CAT4* transcript levels were previously shown to be elevated in *Conyza* following paraquat treatment [22]. However, in the current study, we found no significant changes in *CAT4* transcript levels in response to paraquat treatment (Fig 3). Although the association between *CAT4* and paraquat transport has not previously been reported, another sub-family of polyamine transporters, the L-amino acid transporters, has been shown to play a role in paraquat transport [55, 56]. It is important to note that none of the L-amino acid transporters were reported to be localized to the tonoplast.

Taken together these results do not provide any indication of the involvement of *EPSPS* or *CAT4* genes in glyphosate or paraquat resistance in *Conyza* spp. from California within the first six hours after treatment for paraquat or 24 hours for glyphosate.

We also found that paraquat treatment affected the transcript levels of ABC transporters in *Conyza* spp. *M10* and *M11* transcript levels were higher in all *C*. *bonariensis* lines following paraquat treatment (Fig 3A), while in *C*. *canadensis*, *M11* transcript levels were higher in GPS lines, but not in either of the resistant lines (Fig 3B). However, because these changes in expression were observed in both resistant and susceptible lines, they are unlikely to be associated with the resistance phenotype and are indicative of a more general stress response (Fig 2A and 2B). Changes in expression in response to abiotic stresses, including glyphosate stress, have been documented previously, for example in *Arabidopsis*, where glyphosate stress results in changes in transcript levels of several ABC transporter genes [57, 58].

The results presented establish no association between paraquat resistance and two members of the ABC transporter superfamily, *M10* and *M11*. These findings are consistent with previous studies, which were recently reviewed [18]. Nonetheless, the involvement of ABC transporters cannot be confirmed or discarded based on these data, since only two putative genes were evaluated out of the large ABC transporter gene family. For instance, a transcriptome profiling study of the monocot weed *Eleusine indica* L., reported the transcript levels of many transporters, including members of the ABC transporter family, after paraquat treatment; however, only a small subset of up-regulated genes were associated with paraquat-resistant lines [59]. ABC transporters are known to transport paraquat and have been associated with paraquat resistance in other species. For example, in *Arabidopsis*, loss-of-function mutations in *AtPDR11*, which encodes an ABC-transporter, reduces cellular uptake of paraquat and confers resistance to this herbicide [60].

To aid in the identification of candidate genes associate with resistance mechanism, future studies could benefit from an approach combining transcriptome analysis and forward genetics, an example of which was recently described in *Lolium rigidum* [61]. In this study, a non-targeted approach in diclofop resistant and susceptible lines first to identify changes in expression which were associated with resistance and then to validate their findings using a segregating F_2_ population [58]. Using this approach, candidate genes were identified which were strongly associated with the resistance phenotype [61]. It is also important to note that herbicide resistance phenotypes can result from other changes in gene regulation, such as at the translational or post-translation level. These analyses were beyond the scope of the current study.

## Supporting information

S1 Fig Functional activity of leaves measure by dark adapted chlorophyll fluorescence. Glyphosate-paraquat resistant (GPR) and glyphosate-paraquat-susceptible (GPS) were monitored up to 48 hours after foliar treatment with 9.7 mM of the paraquat cation, equivalent to a field rate of 0.5 kg of paraquat ha^-1^.
(PDF)

S2 Fig Primer specificity test. Melting curves generated for *ACT7* (A, B), *TUA6* (C, D), *eEF1α* (E, F), *eIF4α* (G, H), *GADPH* (I, J), *HPS70* (K, L), *UBQ3* (M, N), *CYP5* (O, P), *CAT4* (Q, R), *M10* (S, T), *M11* (U, V), *EPSPS* (W, X) in *C*. *bonariensis* and *C*. *canadensis*, respectively.
(PDF)

S1 Appendix Consensus sequencing of *Conyza bonariensis* cDNA *M10* and *M11*. Direct sequencing of *M10* and *M11* product using primers developed by Peng et al. 2010. Sequences highlighted in grey indicate introns.
(PDF)

S1 Dataset Cycle threshold (Ct) means and standard error (SE) of reference genes and target genes for each Conyza species, lines, and herbicides tested. N is the number of biological replicates tested. Genes evaluated were *ACTIN 7* (*ACT7*), *TUBULIN ALPHA-6* (*TUA6*), *EUKARYOTIC ELONGATION FACTOR 1-Α* (*eEF-1Α*), *EUKARYOTIC INITIATION FACTOR 4Α* (*eIF-4Α*), *GLYCERALDEHYDE-3-PHOSPHATE DEHYDROGENASE* (*GAPDH*), *HEAT SHOCK PROTEIN 70–4* (*HSP70*), *UBIQUITIN 3* (*UBQ3*), *CYCLOPHILIN 5* (*CYP5*), *ABC-C FAMILY MRP10* (*M10*), *ABC-C FAMILY MRP8* (*M11*), *CATIONIC AMINO ACID TRANSPORTER 4* (*CAT4*), and *5-ENOL-PYRUVYLSHIKIMATE-3-PHOSPHATE SYNTHASE* (*EPSPS*).
(PDF)
