# Supplementary material for: Transcription of putative tonoplast transporters in response to glyphosate and paraquat stress in Conyza bonariensis and Conyza canadensis and selection of reference genes for qRT-PCR
Source: PLoS One. 2017 Jul 10;12(7):e0180794. doi: 10.1371/journal.pone.0180794 (PMC5507266; doi:10.1371/journal.pone.0180794)
Supplement: S1 Fig — Glyphosate-paraquat resistant (GPR) and glyphosate-paraquat-susceptible (GPS) were monitored up to 48 hours after foliar treatment with 9.7 mM of the paraquat cation, equivalent to a field rate of 0.5 kg of paraquat ha-1. (PDF) [file pone.0180794.s001.pdf]

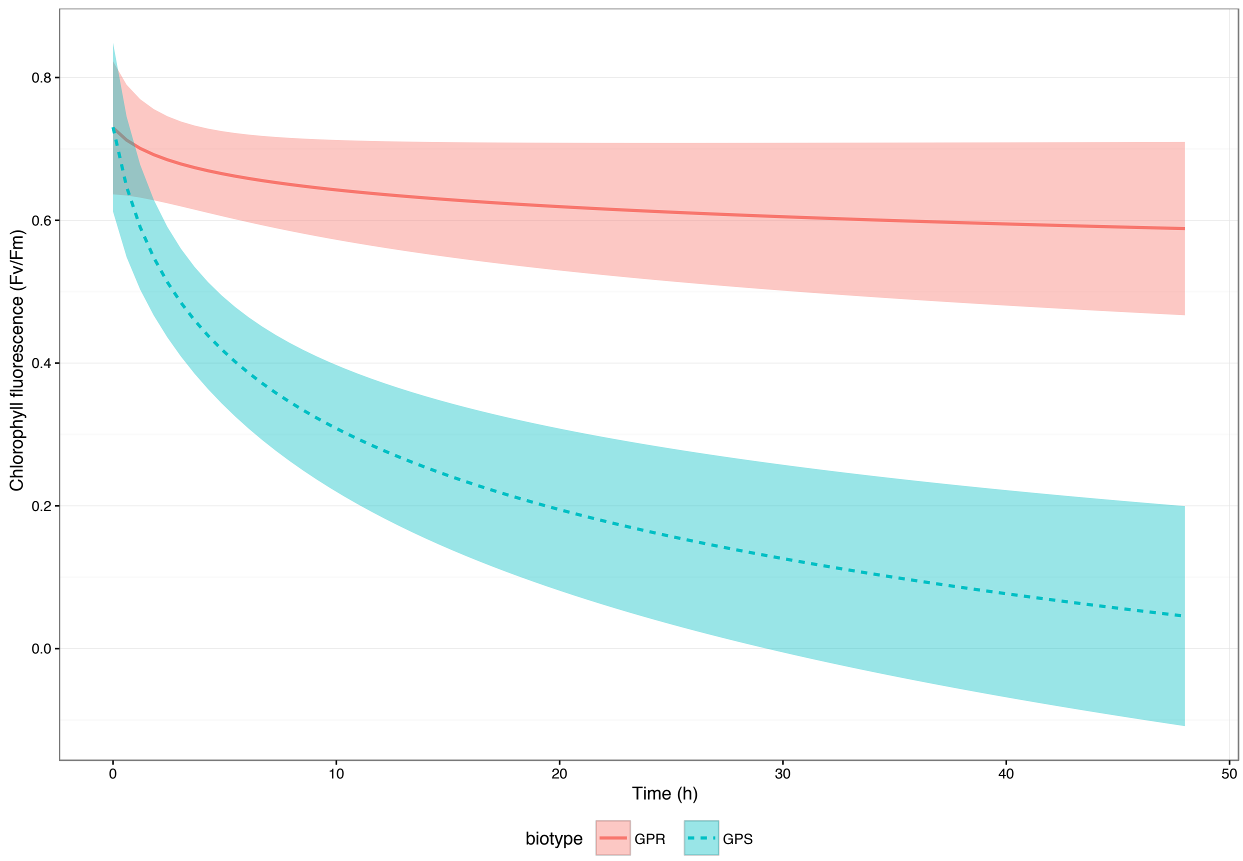

S1 Figure – Functional activity of leaves measure by dark adapted chlorophyll fluorescence.

Glyphosate-paraquat resistant (GPR) and glyphosate-paraquat-susceptible (GPS) were monitored up to 48 hours after foliar treatment with 9.7 mM of the paraquat cation, equivalent to a field rate of 0.5 kg of paraquat ha^{-1}.
