# Supplementary material for: Transcription of putative tonoplast transporters in response to glyphosate and paraquat stress in Conyza bonariensis and Conyza canadensis and selection of reference genes for qRT-PCR
Source: PLoS One. 2017 Jul 10;12(7):e0180794. doi: 10.1371/journal.pone.0180794 (PMC5507266; doi:10.1371/journal.pone.0180794)
Supplement: S2 Fig — Melting curves generated for ACT7 (A, B), TUA6 (C, D), eEF1α (E, F), eIF4α (G, H), GADPH (I, J), HPS70 (K, L), UBQ3 (M, N), CYP5 (O, P), CAT4 (Q, R), M10 (S, T), M11 (U, V), EPSPS (W, X) in C. bonariensis and C. canadensis, respectively. (PDF) [file pone.0180794.s002.pdf]

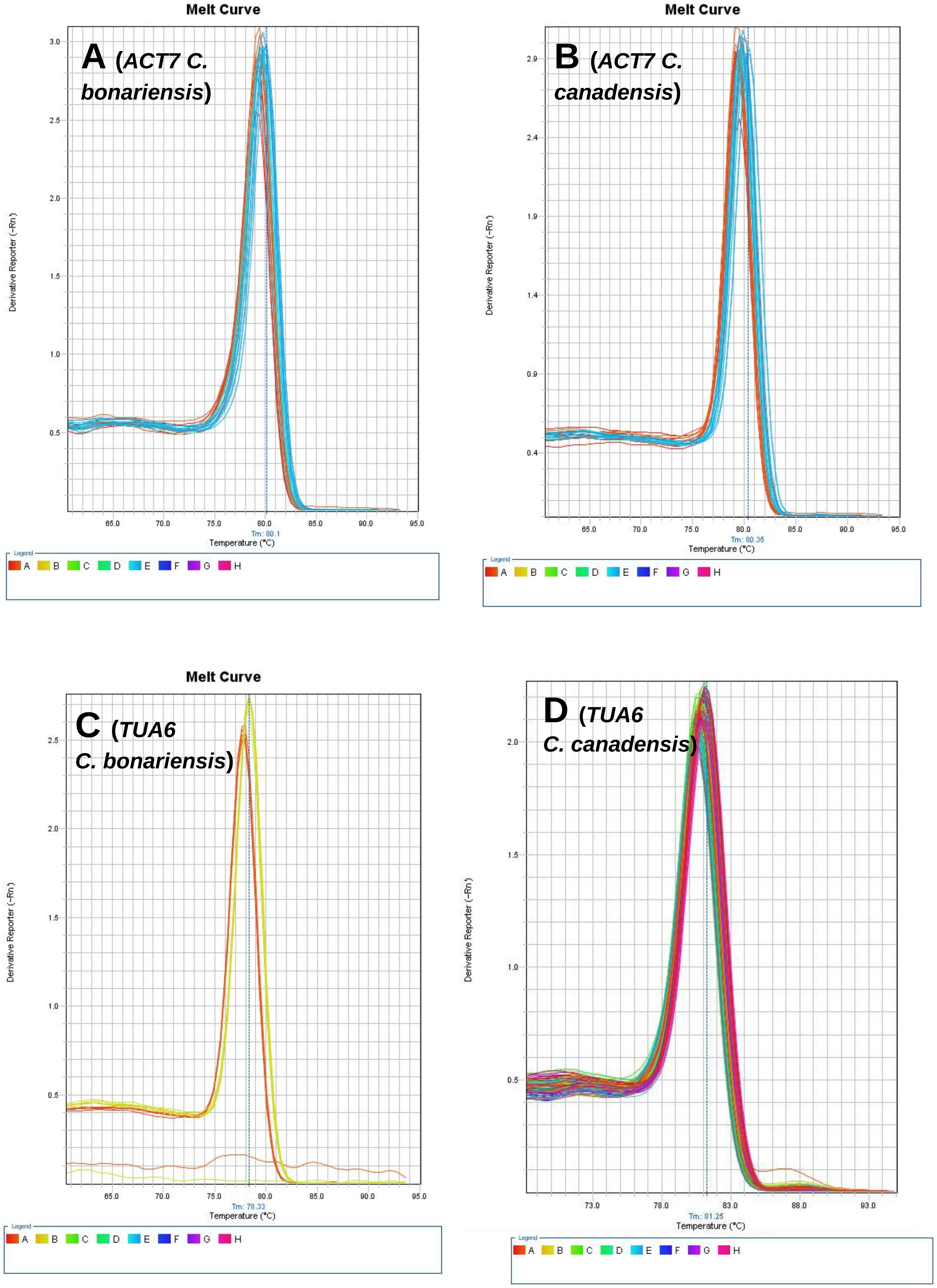
Melt Curve
A (ACT7 C. bonariensis)
Derivative Reporter (-Rn')
Temperature (°C)
Tm: 80.1
Legend
A B C D E F G H
Melt Curve
B (ACT7 C. canadensis)
Derivative Reporter (-Rn')
Temperature (°C)
Tm: 80.35
Legend
A B C D E F G H
Melt Curve
C (TUA6 C. bonariensis)
Derivative Reporter (-Rn')
Temperature (°C)
Tm: 78.33
Legend
A B C D E F G H
D (TUA6 C. canadensis)
Derivative Reporter (-Rn')
Temperature (°C)
Tm: 81.25
Legend
A B C D E F G H

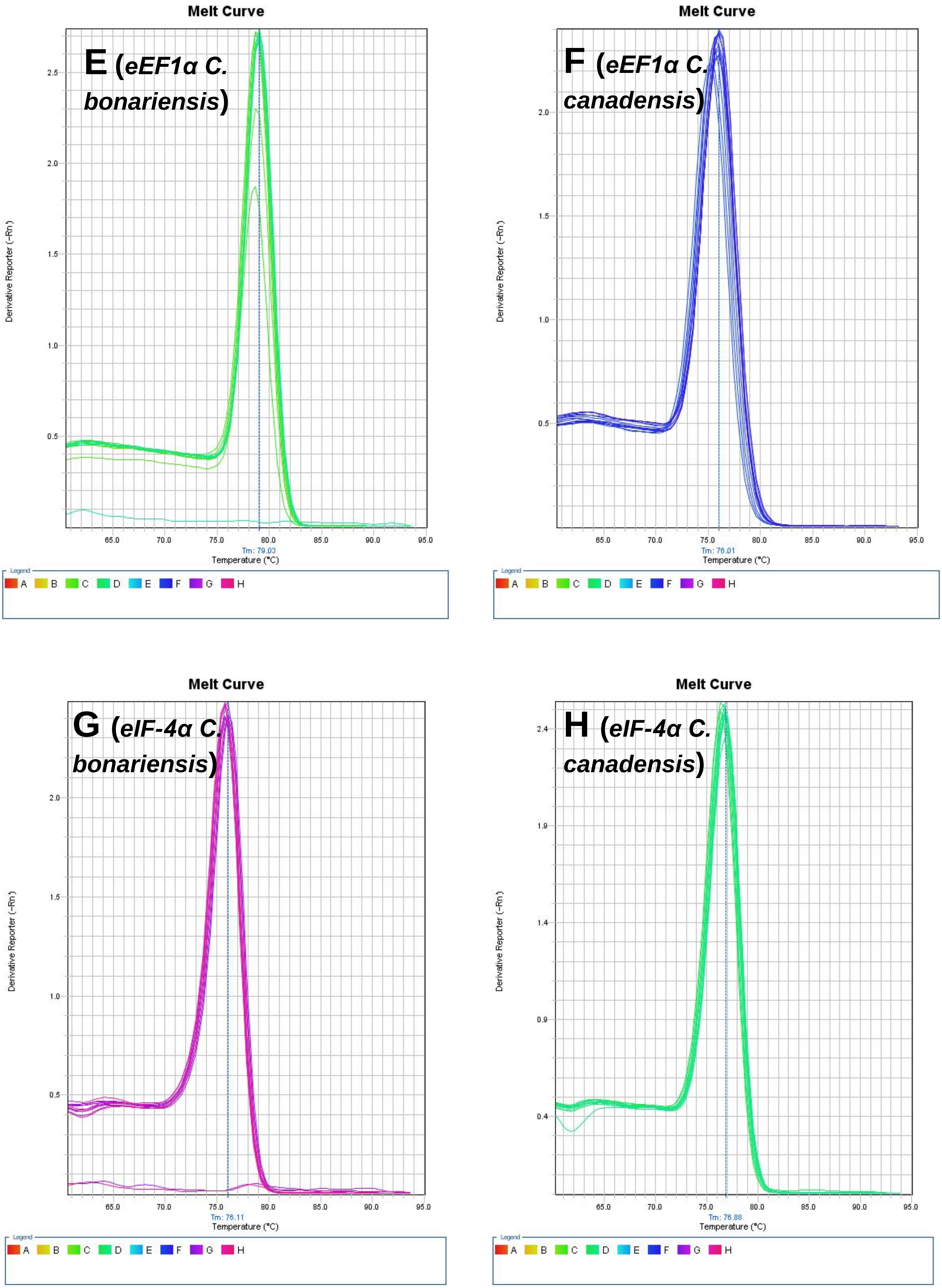

Melt Curve
E (eEF1α C. bonariensis)
Derivative Reporter (−Rn')
Temperature (°C)
Tm: 79.03
Legend
A B C D E F G H
Melt Curve
F (eEF1α C. canadensis)
Derivative Reporter (−Rn')
Temperature (°C)
Tm: 76.01
Legend
A B C D E F G H
Melt Curve
G (eIF-4α C. bonariensis)
Derivative Reporter (−Rn')
Temperature (°C)
Tm: 76.11
Legend
A B C D E F G H
Melt Curve
H (eIF-4α C. canadensis)
Derivative Reporter (−Rn')
Temperature (°C)
Tm: 76.88
Legend
A B C D E F G H

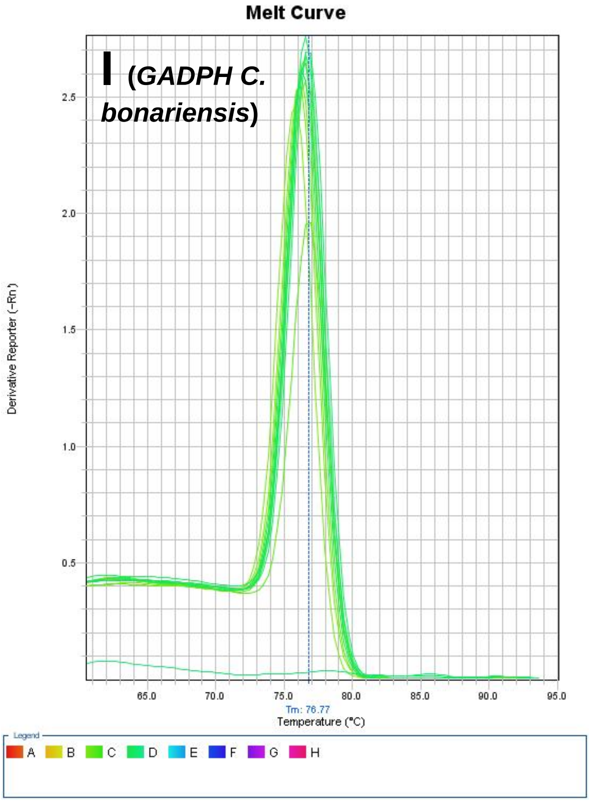
Melt Curve
I (GADPH C. bonariensis)
Derivative Reporter (-Rn')
2.5
2.0
1.5
1.0
0.5
65.0
70.0
75.0
80.0
85.0
90.0
95.0
Tm: 76.77
Temperature (°C)
Legend
A
B
C
D
E
F
G
H

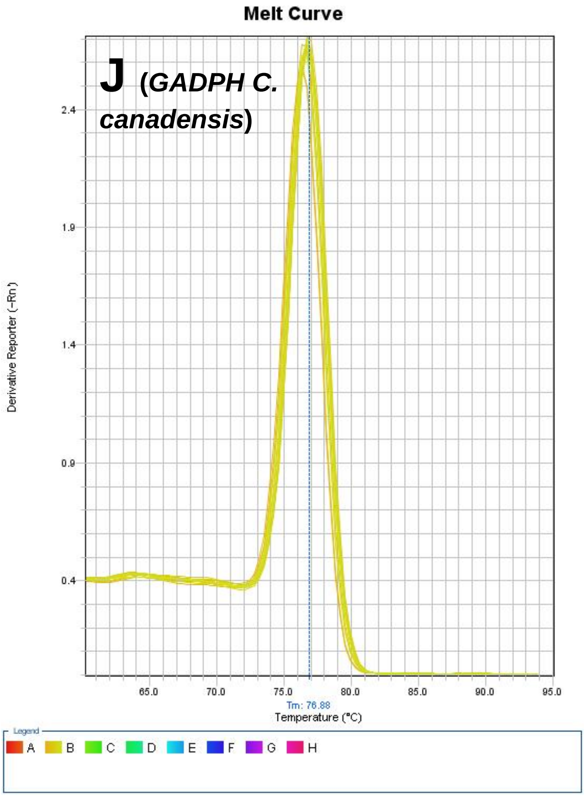
Melt Curve
J (GADPH C. canadensis)
Derivative Reporter (-Rn')
2.4
1.9
1.4
0.9
0.4
65.0
70.0
75.0
80.0
85.0
90.0
95.0
Tm: 76.88
Temperature (°C)
Legend
A
B
C
D
E
F
G
H

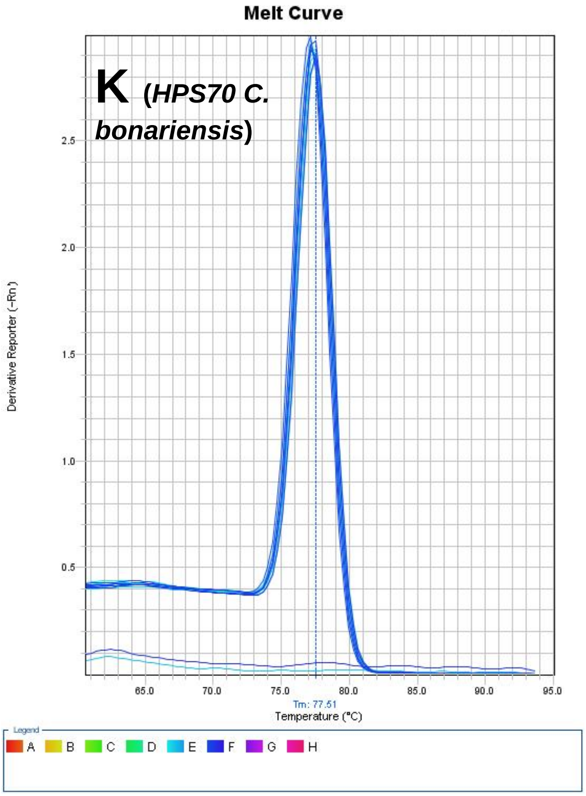
Melt Curve
K (HPS70 C. bonariensis)
Derivative Reporter (-Rn')
2.5
2.0
1.5
1.0
0.5
65.0
70.0
75.0
80.0
85.0
90.0
95.0
Tm: 77.51
Temperature (°C)
Legend
A
B
C
D
E
F
G
H

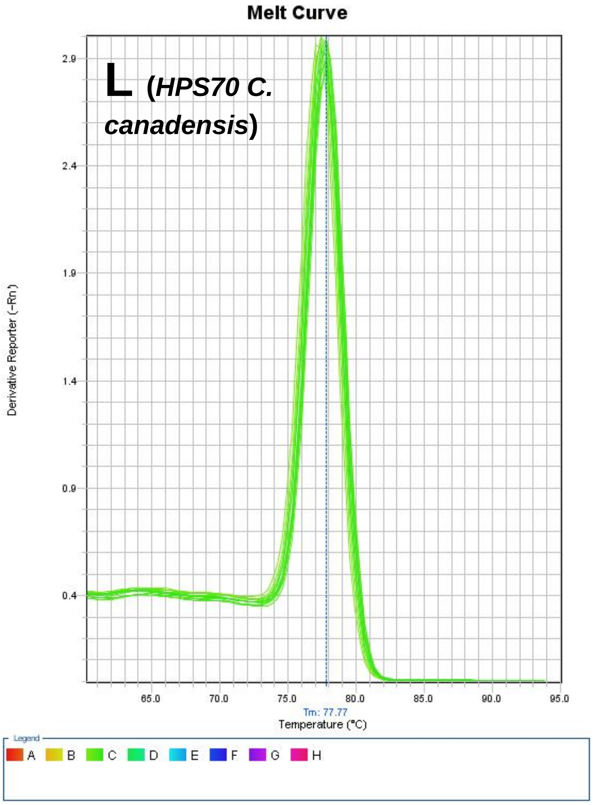
Melt Curve
L (HPS70 C. canadensis)
Derivative Reporter (-Rn')
2.9
2.4
1.9
1.4
0.9
0.4
65.0
70.0
75.0
80.0
85.0
90.0
95.0
Tm: 77.77
Temperature (°C)
Legend
A
B
C
D
E
F
G
H

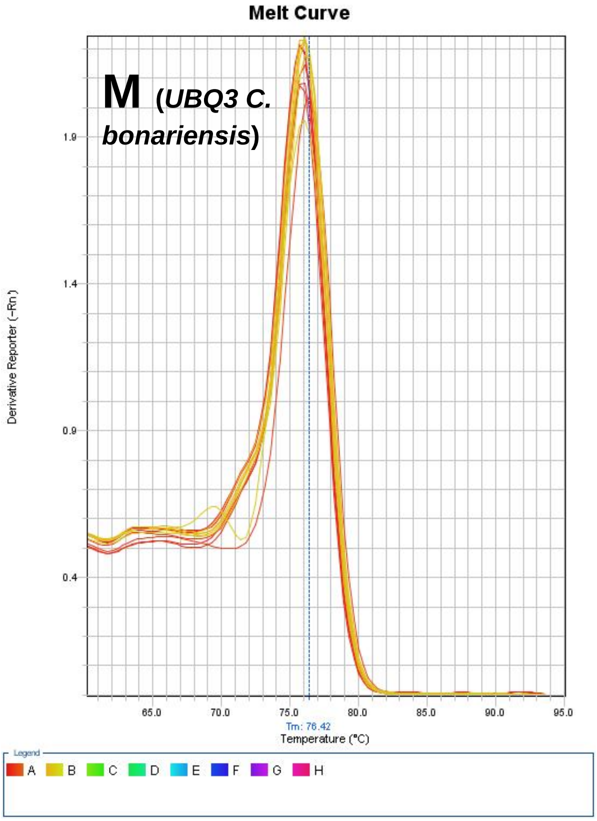
Melt Curve
M (UBQ3 C. bonariensis)
Derivative Reporter (-Rn')
1.9
1.4
0.9
0.4
65.0
70.0
75.0
80.0
85.0
90.0
95.0
Tm: 76.42
Temperature (°C)
Legend
A B C D E F G H

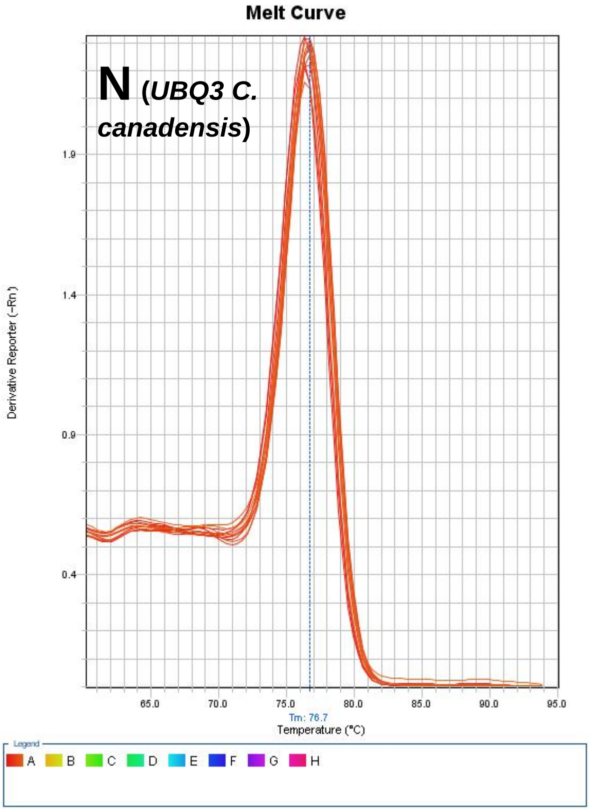
Melt Curve
N (UBQ3 C. canadensis)
Derivative Reporter (-Rn')
1.9
1.4
0.9
0.4
65.0
70.0
75.0
80.0
85.0
90.0
95.0
Tm: 76.7
Temperature (°C)
Legend
A B C D E F G H

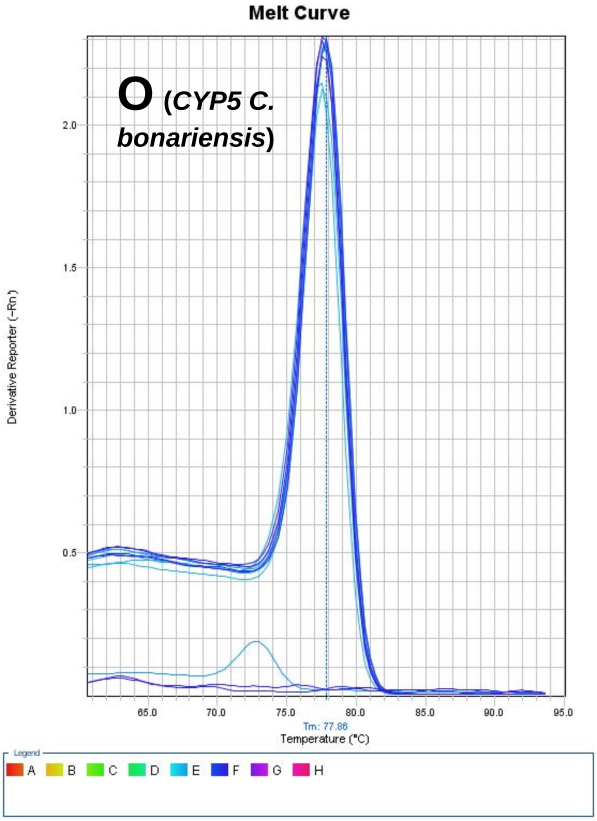
Melt Curve
O (CYP5 C. bonariensis)
Derivative Reporter (-Rn')
2.0
1.5
1.0
0.5
65.0
70.0
75.0
80.0
85.0
90.0
95.0
Tm: 77.86
Temperature (°C)
Legend
A B C D E F G H

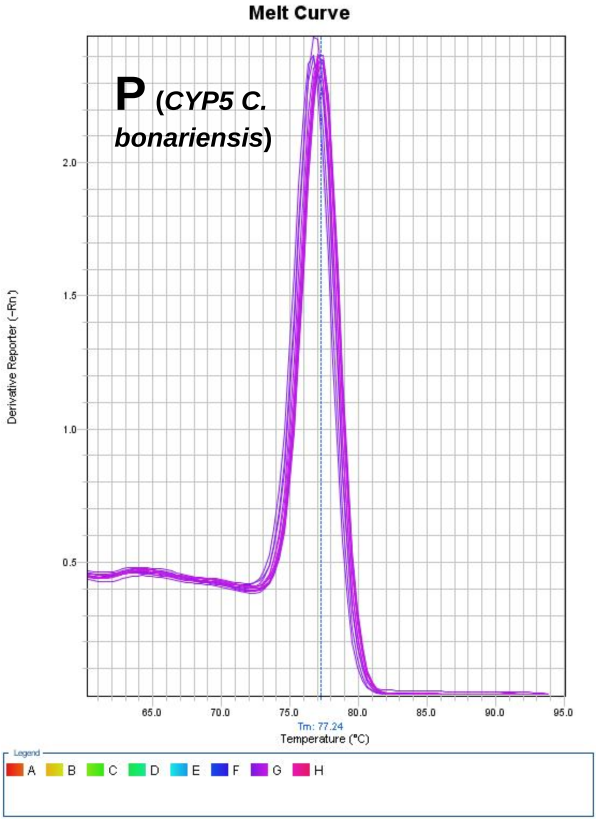
Melt Curve
P (CYP5 C. bonariensis)
Derivative Reporter (-Rn')
2.0
1.5
1.0
0.5
65.0
70.0
75.0
80.0
85.0
90.0
95.0
Tm: 77.24
Temperature (°C)
Legend
A B C D E F G H

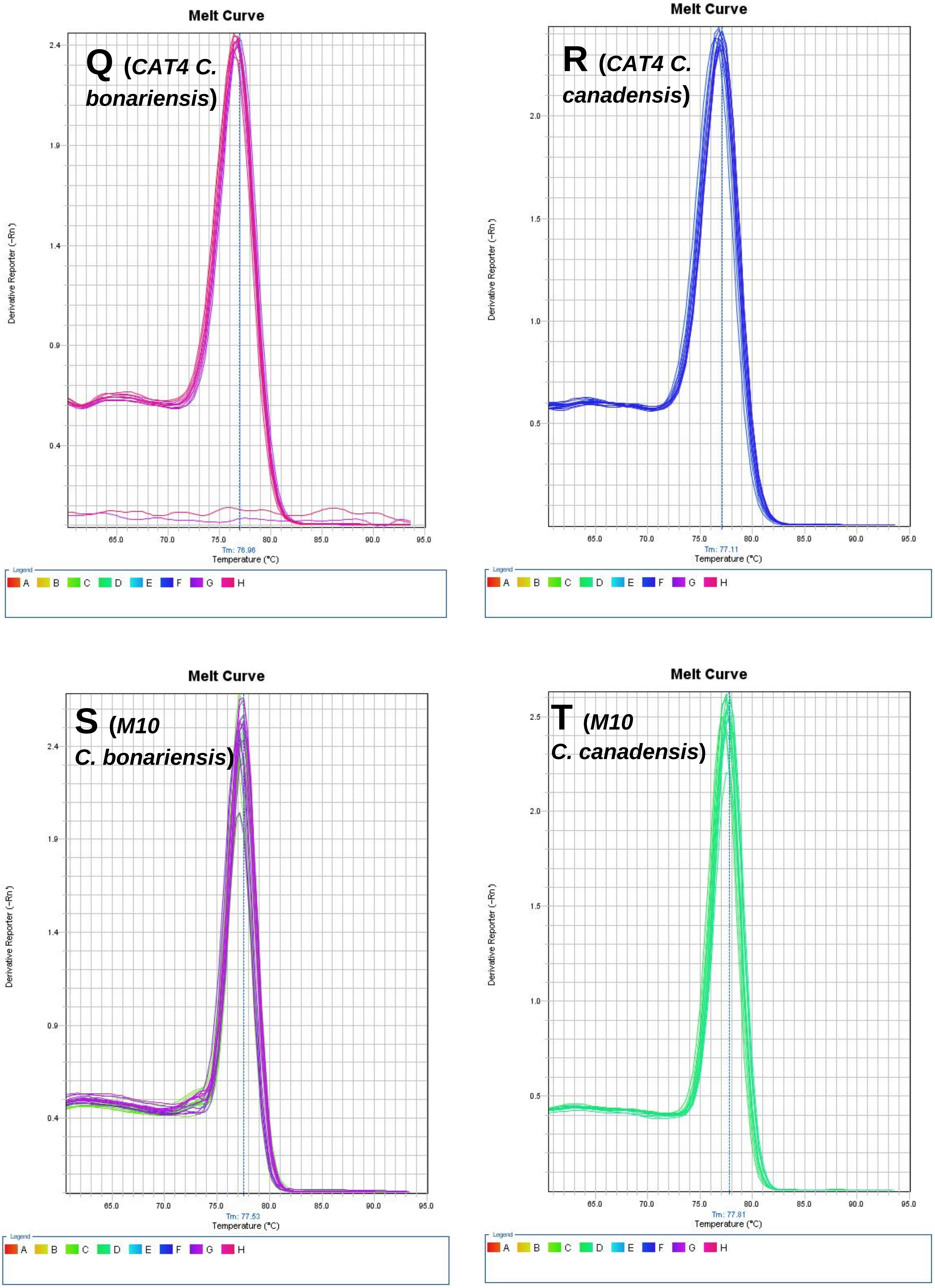
Melt Curve
Q (CAT4 C. bonariensis)
Derivative Reporter (−Rn')
Temperature (°C)
Tm: 76.96
Melt Curve
R (CAT4 C. canadensis)
Derivative Reporter (−Rn')
Temperature (°C)
Tm: 77.11
Melt Curve
S (M10 C. bonariensis)
Derivative Reporter (−Rn')
Temperature (°C)
Tm: 77.53
Melt Curve
T (M10 C. canadensis)
Derivative Reporter (−Rn')
Temperature (°C)
Tm: 77.81
Legend
A B C D E F G H

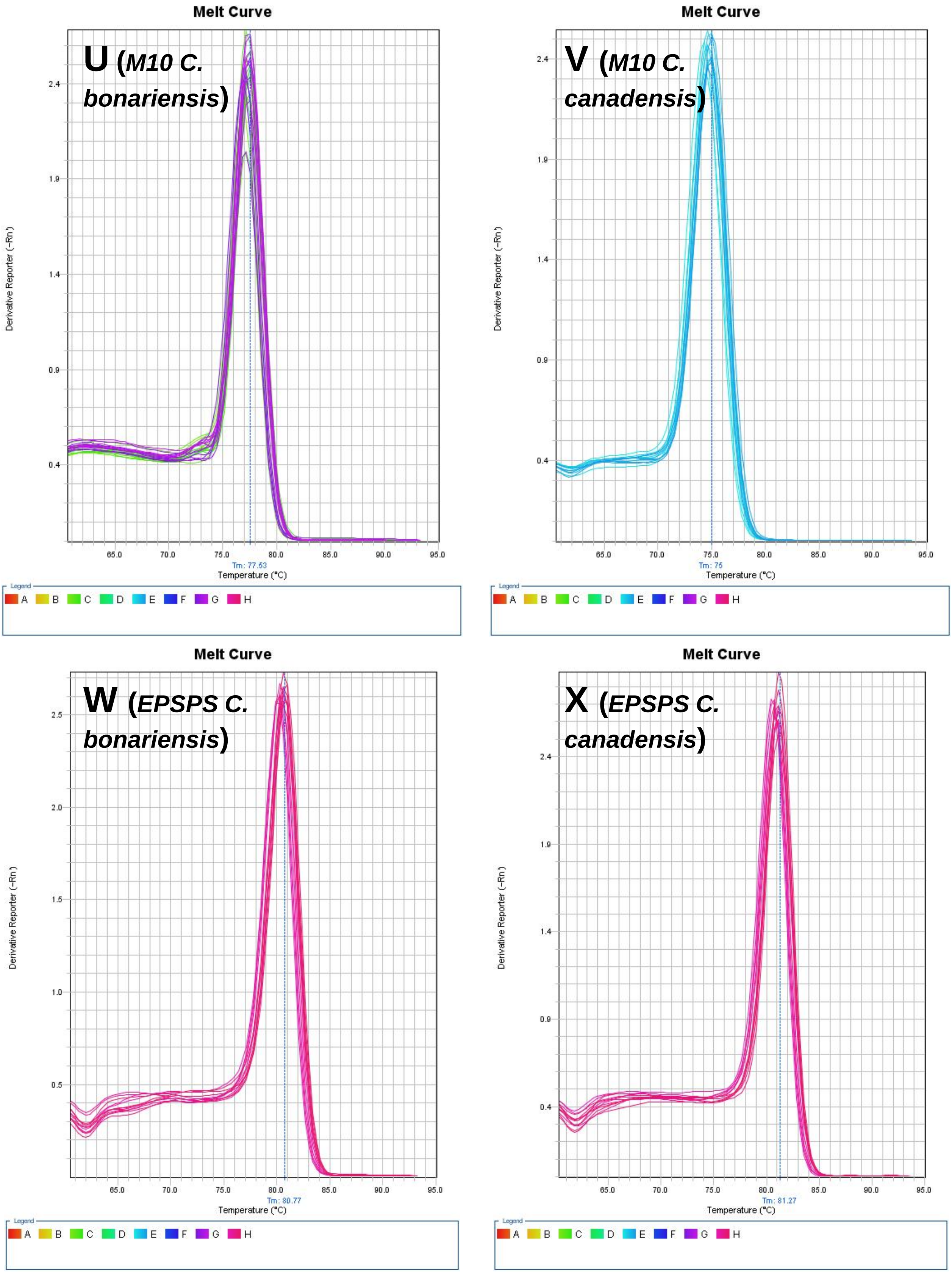

S3 Figure – Primer specificity test. Melting curves generated for *ACT7* (A,B), *TUA6* (C,D), *eEF1α* (E,F), *eIF4α* (G,H), *GADPH* (I,J), *HPS70* (K,L), *UBQ3* (M,N), *CYP5* (O,P), *CAT4* (Q,R), *M10* (S,T), *M11* (U,V), *EPSPS* (W,X), in *C. bonariensis* and *C. canadensis*, respectively.
