# Supplementary material for: Transcription of putative tonoplast transporters in response to glyphosate and paraquat stress in Conyza bonariensis and Conyza canadensis and selection of reference genes for qRT-PCR
Source: PLoS One. 2017 Jul 10;12(7):e0180794. doi: 10.1371/journal.pone.0180794 (PMC5507266; doi:10.1371/journal.pone.0180794)
Supplement: S1 Appendix — Direct sequencing of M10 and M11 product using primers developed by Peng et al. 2010. Sequences highlighted in grey indicate introns. (PDF) [file pone.0180794.s003.pdf]

S1 Appendix. Consensus sequencing of Conyza bonariensis cDNA M10 and M11. Direct sequencing of M10 and M11 product using primers developed by Peng et al. Sequences highlighted in grey indicate introns.

Consensus sequencing of *Conyza bonariensis* cDNA direct sequencing M10 product

CGTGGTATTGGGCTATTACCTTGCAACATCTCGAGAAATAACGCGACTCGACTCAA
TTACAAAAGCACCCGTCATTCACCACTTCTCTGAGAGCATCTCAGGGGTTATGACCA
TCCGGTGTTTCAAAAAACAGGACAGGTTTGTTCAAGAAAATGTTGACCGTGTAGATG
GAAATCTACGGATGGATTTCCACAACAATGCATCGAACGGGTGGTTAGGGTTCCGCT
TGGAATTTCTTGG

Consensus sequencing of *Conyza bonariensis* cDNA direct sequencing M11 product

TTACCTTTGCTGCATTTCTAATTTTCTTAATTTCTATCCCAGAAGGAACTATAGATCC
AAGTATCGCGGGCTTGGCTGCTACTTACGGTCTTACTTTGAACATGTTACAAGGATG
GGTAGTATGGACTTTAACCAACCTTGAAAACAAAATTATTTCTGTTGAAAGGATATT
TCAGTATTCATCTATCCCCAGCGAACCTCCTCTAGTTATAGAATCTAATAGGCCTGAT
GATCAGTGGCCGTCACAGGGAGAAGTTGATATCCGTAACCTGCAGGTTCGGTATGC
ACCACATATGCCACTTGTGTTGCGAGGCCTTACGTGCAA
