# Supplementary material for: Transcription of putative tonoplast transporters in response to glyphosate and paraquat stress in Conyza bonariensis and Conyza canadensis and selection of reference genes for qRT-PCR
Source: PLoS One. 2017 Jul 10;12(7):e0180794. doi: 10.1371/journal.pone.0180794 (PMC5507266; doi:10.1371/journal.pone.0180794)
Supplement: S1 Dataset — N is the number of biological replicates tested. Genes evaluated were ACTIN 7 (ACT7), TUBULIN ALPHA-6 (TUA6), EUKARYOTIC ELONGATION FACTOR 1-Α (eEF-1Α), EUKARYOTIC INITIATION FACTOR 4Α (eIF-4Α), GLYCERALDEHYDE-3-PHOSPHATE DEHYDROGENASE (GAPDH), HEAT SHOCK PROTEIN 70–4 (HSP70), UBIQUITIN 3 (UBQ3), CYCLOPHILIN 5 (CYP5), ABC-C FAMILY MRP10 (M10), ABC-C FAMILY MRP8 (M11), CATIONIC AMINO ACID TRANSPORTER 4 (CAT4), and 5-ENOL-PYRUVYLSHIKIMATE-3-PHOSPHATE SYNTHASE (EPSPS). (PDF) [file pone.0180794.s004.pdf]

S1 Dataset. Cycle threshold (Ct) means and standard error (SE) of reference genes and target genes for each *Conyza* species, lines, and herbicides tested. N is the number of biological replicates tested. Genes evaluated were *ACTIN 7* (*ACT7*), *TUBULIN ALPHA-6* (*TUA6*), *EUKARYOTIC ELONGATION FACTOR 1-A* (*eEF-1A*), *EUKARYOTIC INITIATION FACTOR 4A* (*eIF-4A*), *GLYCERALDEHYDE-3-PHOSPHATE DEHYDROGENASE* (*GAPDH*), *HEAT SHOCK PROTEIN 70-4* (*HSP70*), *UBIQUITIN 3* (*UBQ3*), *CYCLOPHILIN 5* (*CYP5*), *ABC-C FAMILY MRP10* (*M10*), *ABC-C FAMILY MRP8* (*M11*), *CATIONIC AMINO ACID TRANSPORTER 4* (*CAT4*), and *5-ENOL-PYRUVYLSHIKIMATE-3-PHOSPHATE SYNTHASE* (*EPSPS*).

Species	Herbicide	Line	Gene description	N	treated		untreated	
					Ct	SE	Ct	SE
C. bonariensis	glyphosate	GPR	*ACT7*	6	26.1	1.2	25.2	0.6
C. bonariensis	glyphosate	GPR	*TUA6*	3	31.7	2.2	32.7	4.1
C. bonariensis	glyphosate	GPR	*CAT4*	6	30.4	0.7	31.4	1.2
C. bonariensis	glyphosate	GPR	*CYP*	6	22.3	0.7	22.6	0.5
C. bonariensis	glyphosate	GPR	*eEF-1A*	3	26.7	2.9	24.6	2.4
C. bonariensis	glyphosate	GPR	*eIF-4A*	3	27.1	3.7	24.1	2.2
C. bonariensis	glyphosate	GPR	*EPSPS*	6	28.4	1.4	27.8	0.7
C. bonariensis	glyphosate	GPR	*GADPH*	3	23.5	0.3	22.0	2.5
C. bonariensis	glyphosate	GPR	*HPS70*	6	24.5	1.9	24.5	1.2
C. bonariensis	glyphosate	GPR	*M10*	6	27.7	1.3	31.4	0.5
C. bonariensis	glyphosate	GPR	*M11*	6	26.3	1.3	30	0.6
C. bonariensis	glyphosate	GPR	*UBQ3*	3	23.8	1.5	25.0	1.2
C. bonariensis	glyphosate	GPS	*TUA6*	3	34.8	1.5	36.8	1.9
C. bonariensis	glyphosate	GPS	*eEF-1A*	3	27.8	2.5	29.4	2.2
C. bonariensis	glyphosate	GPS	*eIF-4A*	3	29.6	3.1	31.6	2.0
C. bonariensis	glyphosate	GPS	*GADPH*	3	23.7	1.9	25.3	1.8
C. bonariensis	glyphosate	GPS	*UBQ3*	3	25.2	1.5	28.7	1.6
C. bonariensis	glyphosate	GR	*ACT7*	6	26.2	0.8	27.1	0.6
C. bonariensis	glyphosate	GR	*TUA6*	3	33.8	0.9	31.5	0.4
C. bonariensis	glyphosate	GR	*CAT4*	6	31.4	0.6	31.9	0.7
C. bonariensis	glyphosate	GR	*CYP*	6	22.9	0.5	24.2	0.7
C. bonariensis	glyphosate	GR	*eEF-1A*	3	26.4	1.6	27.5	2.2
C. bonariensis	glyphosate	GR	*eIF-4A*	3	26.5	2.7	28.1	0.9
C. bonariensis	glyphosate	GR	*EPSPS*	6	28.5	0.5	29.4	1.3
C. bonariensis	glyphosate	GR	*GADPH*	3	23.4	1.1	23.2	1.8
C. bonariensis	glyphosate	GR	*HPS70*	6	25.6	1.5	26.3	1.2
C. bonariensis	glyphosate	GR	*M10*	6	29.6	0.9	34.8	0.7
C. bonariensis	glyphosate	GR	*M11*	6	27.6	0.9	31.8	0.7
C. bonariensis	glyphosate	GR	*UBQ3*	3	23.3	0.9	27.4	1.0
C. bonariensis	glyphosate	GS	*ACT7*	6	27.4	1.5	28.1	1.4
C. bonariensis	glyphosate	GS	*CAT4*	6	31.2	0.8	32.7	1
C. bonariensis	glyphosate	GS	*CYP*	6	24	1.2	25.2	1.1
C. bonariensis	glyphosate	GS	*EPSPS*	6	31.1	1.9	31	1.2
C. bonariensis	glyphosate	GS	*HPS70*	6	26.7	2	28.2	2.1
C. bonariensis	glyphosate	GS	*M10*	6	28.1	1.4	32.9	1.4

C. bonariensis	glyphosate	GS	*M11*	6	26	1.3	31.7	1.3
C. bonariensis	paraquat	GPR	*ACT7*	6	25.1	0.3	26.1	0.6
C. bonariensis	paraquat	GPR	*TUA6*	3	28.3	2.0	27.6	2.5
C. bonariensis	paraquat	GPR	*CAT4*	6	30.5	0.6	31.1	0.5
C. bonariensis	paraquat	GPR	*CYP*	6	22.5	0.4	23.5	0.4
C. bonariensis	paraquat	GPR	*eEF-1A*	3	24.0	0.8	23.4	1.0
C. bonariensis	paraquat	GPR	*eIF-4A*	3	23.9	0.5	23.6	0.6
C. bonariensis	paraquat	GPR	*EPSPS*	6	26.0	1.2	27.4	1.1
C. bonariensis	paraquat	GPR	*GADPH*	3	22.8	0.7	22.4	0.3
C. bonariensis	paraquat	GPR	*HPS70*	6	24.0	0.3	24.7	0.5
C. bonariensis	paraquat	GPR	*M10*	6	29.8	0.6	32.0	0.5
C. bonariensis	paraquat	GPR	*M11*	6	28.3	0.6	30.3	0.4
C. bonariensis	paraquat	GPR	*UBQ3*	3	25.6	0.8	24.9	0.9
C. bonariensis	paraquat	GPS	*TUA6*	3	29.6	2.6	24.9	1.6
C. bonariensis	paraquat	GPS	*eEF-1A*	3	25.3	1.7	21.8	1.0
C. bonariensis	paraquat	GPS	*eIF-4A*	3	26.3	1.5	22.0	0.3
C. bonariensis	paraquat	GPS	*GADPH*	3	24.7	0.5	22.0	0.6
C. bonariensis	paraquat	GPS	*UBQ3*	3	27.0	1.5	24.9	0.1
C. bonariensis	paraquat	GR	*ACT7*	6	27.2	0.5	25.4	0.4
C. bonariensis	paraquat	GR	*TUA6*	3	29.2	1.8	26.4	1.6
C. bonariensis	paraquat	GR	*CAT4*	6	31.3	0.2	29.6	0.6
C. bonariensis	paraquat	GR	*CYP*	6	23.6	0.4	22.9	0.4
C. bonariensis	paraquat	GR	*eEF-1A*	3	24.8	0.0	23.0	0.7
C. bonariensis	paraquat	GR	*eIF-4A*	3	24.4	0.4	23.1	0.6
C. bonariensis	paraquat	GR	*EPSPS*	6	28.3	1.2	26.8	1.0
C. bonariensis	paraquat	GR	*GADPH*	3	23.7	1.3	21.9	0.4
C. bonariensis	paraquat	GR	*HPS70*	6	24.7	0.4	23.5	0.5
C. bonariensis	paraquat	GR	*M10*	6	31.0	0.5	31.9	0.5
C. bonariensis	paraquat	GR	*M11*	6	28.2	0.4	29.6	0.6
C. bonariensis	paraquat	GR	*UBQ3*	3	26.5	0.8	25.1	0.4
C. bonariensis	paraquat	GS	*ACT7*	6	28.5	0.9	24.2	0.3
C. bonariensis	paraquat	GS	*CAT4*	6	32.9	1.0	28.8	0.4
C. bonariensis	paraquat	GS	*CYP*	6	25.4	1.0	21.8	0.2
C. bonariensis	paraquat	GS	*EPSPS*	6	30.2	1.8	25.2	0.9
C. bonariensis	paraquat	GS	*HPS70*	6	26.6	1.2	22.0	0.4
C. bonariensis	paraquat	GS	*M10*	6	31.5	1.2	28.8	0.7
C. bonariensis	paraquat	GS	*M11*	6	29.2	1.2	27.7	0.5
C. canadensis	glyphosate	GPR	*ACT7*	5	24.8	0.1	24.5	0.3
C. canadensis	glyphosate	GPR	*TUA6*	3	28.8	0.4	27.5	0.6
C. canadensis	glyphosate	GPR	*CAT4*	5	29.8	0.4	30.0	0.3
C. canadensis	glyphosate	GPR	*CYP*	5	22.1	0.1	22.4	0.1
C. canadensis	glyphosate	GPR	*eEF-1A*	3	22.8	0.4	23.4	0.2
C. canadensis	glyphosate	GPR	*eIF-4A*	3	23.3	0.8	23.3	0.7
C. canadensis	glyphosate	GPR	*EPSPS*	5	27.2	0.2	27.5	0.2

C. canadensis	glyphosate	GPR	*GADPH*	3	20.9	0.5	19.6	0.4
C. canadensis	glyphosate	GPR	*HPS70*	5	21.4	0.4	21.9	0.3
C. canadensis	glyphosate	GPR	*M10*	5	27.5	0.3	31.7	0.4
C. canadensis	glyphosate	GPR	*M11*	5	27.6	0.4	30.0	0.3
C. canadensis	glyphosate	GPR	*UBQ3*	3	25.3	0.3	26.6	0.4
C. canadensis	glyphosate	GPS	*ACT7*	5	25.0	0.5	24.6	0.2
C. canadensis	glyphosate	GPS	*TUA6*	3	27.9	0.8	27.4	0.5
C. canadensis	glyphosate	GPS	*CAT4*	5	29.8	0.4	29.8	0.2
C. canadensis	glyphosate	GPS	*CYP*	5	22.0	0.7	21.9	0.5
C. canadensis	glyphosate	GPS	*eEF-1A*	3	23.1	0.3	23.3	0.3
C. canadensis	glyphosate	GPS	*eIF-4A*	3	23.7	1.5	23.0	0.7
C. canadensis	glyphosate	GPS	*EPSPS*	5	27.8	0.4	27.7	0.4
C. canadensis	glyphosate	GPS	*GADPH*	3	22.5	0.7	20.9	0.5
C. canadensis	glyphosate	GPS	*HPS70*	5	21.6	0.2	21.5	0.3
C. canadensis	glyphosate	GPS	*M10*	5	27.0	0.4	31.3	0.3
C. canadensis	glyphosate	GPS	*M11*	5	26.1	0.4	30.1	0.3
C. canadensis	glyphosate	GPS	*UBQ3*	3	25.8	0.9	26.7	0.6
C. canadensis	glyphosate	GR	*ACT7*	5	24.4	0.5	24.6	0.3
C. canadensis	glyphosate	GR	*TUA6*	3	28.1	0.5	27.2	0.7
C. canadensis	glyphosate	GR	*CAT4*	5	29.6	0.4	29.9	0.2
C. canadensis	glyphosate	GR	*CYP*	5	22.1	0.5	22.3	0.2
C. canadensis	glyphosate	GR	*eEF-1A*	3	22.5	0.3	23.6	0.4
C. canadensis	glyphosate	GR	*eIF-4A*	3	22.9	1.0	23.5	0.7
C. canadensis	glyphosate	GR	*EPSPS*	5	27.5	0.4	28.0	0.1
C. canadensis	glyphosate	GR	*GADPH*	3	20.6	0.3	19.5	0.4
C. canadensis	glyphosate	GR	*HPS70*	5	21.6	0.6	21.5	0.3
C. canadensis	glyphosate	GR	*M10*	5	26.7	0.6	31.9	0.7
C. canadensis	glyphosate	GR	*M11*	5	25.8	0.6	30.0	0.3
C. canadensis	glyphosate	GR	*UBQ3*	3	24.8	0.5	26.6	0.4
C. canadensis	paraquat	GPR	*ACT7*	5	24.7	0.6	24.8	0.4
C. canadensis	paraquat	GPR	*TUA6*	3	28.3	0.7	26.0	1.1
C. canadensis	paraquat	GPR	*CAT4*	5	29.4	0.6	30.4	0.7
C. canadensis	paraquat	GPR	*CYP*	5	22.6	0.6	22.9	0.4
C. canadensis	paraquat	GPR	*eEF-1A*	3	23.7	0.7	22.2	1.4
C. canadensis	paraquat	GPR	*eIF-4A*	3	24.1	0.7	22.6	0.7
C. canadensis	paraquat	GPR	*EPSPS*	5	27.1	0.9	27.6	0.4
C. canadensis	paraquat	GPR	*GADPH*	3	22.1	0.9	19.0	0.7
C. canadensis	paraquat	GPR	*HPS70*	5	22.4	0.7	23.1	0.6
C. canadensis	paraquat	GPR	*M10*	5	29.3	0.5	31.6	0.6
C. canadensis	paraquat	GPR	*M11*	5	28.6	0.4	30.0	0.5
C. canadensis	paraquat	GPR	*UBQ3*	3	26.9	0.4	25.6	0.3
C. canadensis	paraquat	GPS	*ACT7*	5	27.0	0.6	24.0	0.5
C. canadensis	paraquat	GPS	*TUA6*	3	28.0	0.5	27.8	0.7
C. canadensis	paraquat	GPS	*CAT4*	5	31.8	0.6	28.3	0.7

C. canadensis	paraquat	GPS	*CYP*	5	24.2	0.5	21.7	0.5
C. canadensis	paraquat	GPS	*eEF-1A*	3	24.0	1.1	24.0	0.4
C. canadensis	paraquat	GPS	*eIF-4A*	3	24.6	0.3	24.6	0.7
C. canadensis	paraquat	GPS	*EPSPS*	5	29.9	0.7	27.2	0.6
C. canadensis	paraquat	GPS	*GADPH*	3	21.1	0.3	21.5	0.1
C. canadensis	paraquat	GPS	*HPS70*	5	24.2	0.5	22.1	0.5
C. canadensis	paraquat	GPS	*M10*	5	33.1	1.0	30.5	0.7
C. canadensis	paraquat	GPS	*M11*	5	30.2	0.8	29.7	0.7
C. canadensis	paraquat	GPS	*UBQ3*	3	26.1	0.1	27.3	0.3
C. canadensis	paraquat	GR	*ACT7*	5	27.7	0.9	25.4	0.2
C. canadensis	paraquat	GR	*TUA6*	3	33.1	1.8	28.5	0.4
C. canadensis	paraquat	GR	*CAT4*	5	32.6	0.9	30.3	0.5
C. canadensis	paraquat	GR	*CYP*	5	25.0	0.7	23.1	0.2
C. canadensis	paraquat	GR	*eEF-1A*	3	28.8	1.3	24.4	0.3
C. canadensis	paraquat	GR	*eIF-4A*	3	29.8	1.5	24.4	0.5
C. canadensis	paraquat	GR	*EPSPS*	5	31.8	0.9	29.0	0.3
C. canadensis	paraquat	GR	*GADPH*	3	27.2	1.1	20.6	0.3
C. canadensis	paraquat	GR	*HPS70*	5	25.6	1.0	23.1	0.4
C. canadensis	paraquat	GR	*M10*	5	33.4	0.9	31.6	0.7
C. canadensis	paraquat	GR	*M11*	5	30.8	0.6	30.1	0.4
C. canadensis	paraquat	GR	*UBQ3*	3	29.7	1.1	27.4	0.5
